# Use of warfarin in long-term care: a systematic review

**DOI:** 10.1186/1471-2318-12-14

**Published:** 2012-04-05

**Authors:** Marjorie Neidecker, Aarti A Patel, Winnie W Nelson, Gregory Reardon

**Affiliations:** 1 Informagenics, LLC, 450 W. Wilson Bridge Rd., Suite 340, Worthington, OH 43085, USA; 2 The Ohio State University College of Pharmacy, Columbus, OH, USA; 3 Janssen Scientific Affairs, LLC, Raritan, NJ, USA

## Abstract

**Background:**

The use of warfarin in older patients requires special consideration because of concerns with comorbidities, interacting medications, and the risk of bleeding. Several studies have suggested that warfarin may be underused or inconsistently prescribed in long-term care (LTC); no published systematic review has evaluated warfarin use for stroke prevention in this setting. This review was conducted to summarize the body of published original research regarding the use of warfarin in the LTC population.

**Methods:**

A systematic literature search of the PubMed, Cumulative Index to Nursing and Allied Health Literature, and Cochrane Library was conducted from January 1985 to August 2010 to identify studies that reported warfarin use in LTC. Studies were grouped by (1) rates of warfarin use and prescribing patterns, (2) association of resident and institutional characteristics with warfarin prescribing, (3) prescriber attitudes and concerns about warfarin use, (4) warfarin management and monitoring, and (5) warfarin-related adverse events. Summaries of study findings and quality assessments of each study were developed.

**Results:**

Twenty-two studies met the inclusion criteria for this review. Atrial fibrillation (AF) was the most common indication for warfarin use in LTC and use of warfarin for stroke survivors was common. Rates of warfarin use in AF were low in 5 studies, ranging from 17% to 57%. These usage rates were low even among residents with high stroke risk and low bleeding risk. Scored bleeding risk had no apparent association with warfarin use in AF. In physician surveys, factors associated with not prescribing warfarin included risk of falls, dementia, short life expectancy, and history of bleeding. International normalized ratio was in the target range approximately half of the time. The combined overall rate of warfarin-related adverse events and potential events was 25.5 per 100 resident months on warfarin therapy.

**Conclusions:**

Among residents with AF, use of warfarin and maintenance of INR levels to prevent stroke appear to be suboptimal. Among prescribers, perceived challenges associated with warfarin therapy often outweigh its benefits. Further research is needed to explicitly consider the appropriate balancing of risks and benefits in this frail patient population.

## Background

Beginning in the year 2015, the greatest population increases in the United States are expected to occur among persons aged 65 years and older [1]. With increasing age, the risk for developing thromboembolic disease, including deep vein thrombosis (DVT), pulmonary embolism (PE), myocardial infarction (MI), and stroke increases correspondingly. Among stroke survivors or patients with atrial fibrillation (AF), warfarin has been used for prevention of thromboembolic stroke, although among non-cardioembolic stroke survivors without AF, antiplatelet agents are first-line therapy [2]. The use of warfarin in older patients requires special consideration because of comorbidities such as kidney disease and diabetes, the use of multiple and potentially interacting medications, and the risk of bleeding, all of which increase with age and concurrently with age-related diseases [3-6].

A number of studies have suggested that warfarin may be underused or inconsistently prescribed in long-term care (LTC) facilities [7-13]. There are several reasons this may be the case. For one, evidence-based guidelines are not easily developed for the LTC population, and clinical trials measuring the efficacy of warfarin are rarely performed in the LTC setting. Clinicians are thus left with incomplete evidence for generalizing study findings from non-LTC patients to older and more frail LTC residents [14]. Further, clinicians are likely to be concerned about major bleeding in older patients [14,15] and consequently reluctant to prescribe warfarin when indicated.

To objectively reconcile issues of benefit and risk associated with warfarin use in LTC residents and to explore its usage in this setting, a review of the findings from empirical research conducted in this setting is warranted. The purpose of this review was to identify and summarize the body of published original research regarding the use of warfarin in LTC. Because the studies reviewed here differ in purpose, articles are grouped by the most common topics identified: (1) warfarin rates of use and prescribing patterns, (2) association of resident and/or facility characteristics with warfarin prescribing, (3) prescriber attitudes and concerns about warfarin use, (4) warfarin management and monitoring, and (5) warfarin-related adverse events.

## Methods

### Literature search

**Figure 1:**
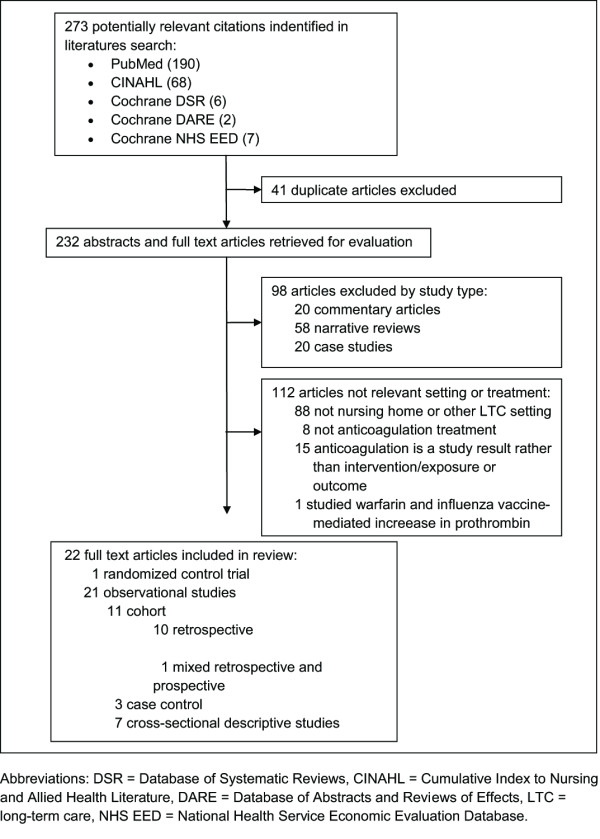
**Literature search and study selection process**.

Two investigators (MN and GR) conducted a systematic search of the PubMed, Cumulative Index to Nursing and Allied Health Literature (CINAHL), Cochrane Database of Systematic Reviews (CDSR), Database of Abstracts and Reviews of Effects (DARE) and National Health Service Economic Evaluation Database (NHS EED) databases (Figure 1). Title, abstract, and text searches for each database were performed for the following search terms: (anticoag* OR warfarin) AND ("long-term care" OR "nursing home"). The search was repeated in PubMed using the Medical Subject Heading (MeSH) database search key words "nursing home" and "long-term care." Additionally, the websites of the Agency for Healthcare Research and Quality (AHRQ) and the American Medical Directors Association (AMDA) were searched. A manual review of references from each pertinent article, review articles, and treatment guidelines was also conducted to identify additional related articles.

### Study selection

Two investigators (MN and GR) independently determined study eligibility, with disagreement resolved by discussion. A study was deemed eligible for inclusion in the systematic review if it: (1) reported on prescribing or use of warfarin alone or in combination with antiplatelet medications in the LTC setting, (2) was published in English, (3) was published between January 1985 and August 2010, and (4) reported original research. Editorials, letters, commentaries, reviews, expert opinions or clinical topic discussions, guidelines, and case studies were excluded from this review.

### Data abstraction

For each included study, data were independently abstracted by two investigators (MN and GR) to include the following details: study objective, intervention/exposure and outcomes, study design, data source, study population, study setting, time period, summary of results, funding source, and quality assessment.

### Quality assessment

Two investigators (MN and GR) compiled lists of strengths and weaknesses in the methodology of each study upon the initial reading. Using the quality assessment methodology for observational studies and randomized controlled trials developed by Williams et al. for the AHRQ [16], one investigator (GR) responded to the 11 structured closed-ended questions regarding quality for each observational study and the 10 structured closed-ended questions for the single randomized controlled trial reviewed.

## Results

### Study identification and characteristics

The initial search of the four electronic databases returned 273 citations, of which 41 were duplicates, leaving 232 citations for abstract and full text review. Upon review, 210 of these were excluded for failing to meet all study selection criteria. Thus, a total of 22 studies [7-13,17-31] were retained. These included: one randomized controlled trial [20]; 10 retrospective cohort studies [8,10,11,18,19,21-23,25,30], one mixed prospective and retrospective cohort study [24], three case-control studies [7,26,31], and seven cross-sectional studies [9,12,13,17,27-29].

Across all 22 studies, the total number of LTC residents studied was 130,757, with sample sizes in individual studies ranging from 37 [31] to 53,829 [12]. Study duration ranged from 1 month [9] to 6 years [26]. Among the included studies, 14 reported on the rate of warfarin prevalence and prescribing patterns [7-13,17-21,24,25], eight evaluated the association of resident or facility characteristics with warfarin prescribing [7,8,10-13,17,18], three reported on prescriber attitudes and concerns regarding warfarin use [27-29], nine discussed warfarin management and monitoring [8,9,11,22-25,30,31], and two reported on warfarin-related adverse events [25,26]. Unless otherwise indicated below, significance was reported at 95% confidence.

### Rate of warfarin use and prescribing patterns

Of the 14 studies (112,754 total residents) that examined the rate of warfarin use or prescribing patterns in LTC facilities, 11 (Table 1 Part A) [7-13,17-19,25] examined residents with various conditions for which warfarin is indicated, comparing residents who were prescribed warfarin with those who were not prescribed warfarin. Three of these studies [20,21,24] examined consultant pharmacist interventions with regard to warfarin prescribing in the facilities studied (Table 1 Part B).

**Table 1:** Warfarin indications, rate of use and prescribing patterns

Study	Study objective, (intervention/exposure and outcomes)	Study design, data source	Study population, study setting, time period	Results	Quality assessment, funding source
**Part A: Warfarin exposure**					

Abdel-Latif et al. (2005) [7]	To determine predictors of OAC therapy for AF in LTC	*Design*: case control study *Data source*: medical chart, pharmacy record, and MDS	*Population*: 117 residents with chronic or paroxysmal AF identified from 934 total residents *Setting*: 6 LTC facilities with > 100 beds in Cleveland metropolitan area (US) *Time period*: not specified	Among 117 residents (12.5% of 934) with AF, OAC was prescribed for 46%; aspirin or clopidogrel: 40%; no antithrombotic treatment: 21%. Logistic regression produced 2 independent predictors of OAC prescription: (1) Prior stroke was the primary determinant of receiving OAC (OR = 0.02; 95% CI = 0.09-0.47) *sic*, and (2) history of GI bleeding was a predictor for *not *receiving OAC (OR, 5.6; 95% CI = 1.1- 29.4). Classification and regression tree analysis found residents with prior stroke or GI bleeding and no history of coronary artery disease and who were non-Caucasian were less likely to be prescribed OAC. Those without stroke were less likely to be prescribed OAC if they were younger, had dementia or lower functional status	*Quality assessment for observational studies:* 1) Unbiased selection of the cohort? Yes 2) Selection minimizes baseline differences in prognostic factors? Yes 3) Sample size calculated/5% difference? No 4) Adequate description of the cohort? Yes 5) Validated method for ascertaining exposure? Yes 6) Validated method for ascertaining clinical outcomes? Yes 7) Outcome assessment blind to exposure? Yes 8) Adequate follow-up period? Yes; date of study not specified; cross-sectional 9) Completeness of follow-up? Yes 10) Analysis controls for confounding? Yes 11) Analytic methods appropriate? Partial; MDS cognition and functional scale scoring methods not referenced; multivariate findings not fully reported *Funding*: not specified

Christian et al. (2003) [17]	To evaluate the extent to which people of color (e.g. non-white or Hispanic) in US nursing homes were less likely to receive pharmacologic treatment of recurrent stroke	*Design*: retrospective cross-sectional study *Data source*: the SAGE database (links inpatient Medicare claims, drug data, and MDS data)	*Population*: 19,051 nursing home residents with recent hospitalization for ischemic stroke *Setting*: Kansas, Maine, Mississippi, Ohio, New York, and South Dakota (US) *Time period*: 1992-1996	Variability in use of any treatment for secondary stroke prevention (warfarin or antiplatelet agent) was observed by race/ethnicity: 58% of American Indians received therapy, 54% of non-Hispanic whites, 49% of non-Hispanic blacks, 46% of Hispanics, and only 39% of Asian/Pacific Islanders. The use of warfarin among residents with conditions warranting anticoagulant therapy was 40% among non-Hispanic whites, 36% among American Indians, 32% among non-Hispanic blacks, 26% of Asian/Pacific Islanders, and 25% among Hispanics. After controlling for confounding, Asian/Pacific Islanders (prevalence difference = -5.2, CI = -18.1 to 7.8), non-Hispanic black residents (prevalence difference = -7.6, CI = -11.2 to -3.9), and Hispanics (prevalence difference = -7.6, CI = -17.6 to 2.2) received warfarin less often than non-Hispanic whites	*Quality assessment for observational studies:* 1) Unbiased selection of the cohort? Yes 2) Selection minimizes baseline differences in prognostic factors? Yes 3) Sample size calculated/5% difference? Partial; likely lacked statistical power for Asian/Pacific Islanders, Hispanics, and American Indians 4) Adequate description of the cohort? Yes 5) Validated method for ascertaining exposure? Yes 6) Validated method for ascertaining clinical outcomes? Yes 7) Outcome assessment blind to exposure? Yes 8) Adequate follow-up period? No; uses data from admission only so secondary stroke prevention was more likely ordered at hospital discharge, not in LTC facility 9) Completeness of follow-up? No 10) Analysis controls for confounding? Yes 11) Analytic methods appropriate? Partial; prevalence differences reported rather than association strength (i.e. no OR) *Funding*: National Institute on Aging, AHRQ

Gurwitz et al. (2007) [25]	To examine the preventability of actual and potential warfarin-related adverse events in the nursing home setting	*Design*: retrospective cohort study *Data source*: nursing home records	*Population*: all 490 residents of 25 nursing homes receiving warfarin therapy *Setting*: 25 nursing homes (bed size range, 90-360) in Connecticut (US) *Time period*: 12- month observation period (Apr 2003 - Mar 2004)	The most common indications for warfarin therapy included stroke prevention in AF (58%), treatment/prevention of DVT or PE (26%), and stroke prevention without AF (12%)	*Quality assessment for observational studies:* 1) Unbiased selection of the cohort? Yes 2) Selection minimizes baseline differences in prognostic factors? Yes 3) Sample size calculated/5% difference? Yes 4) Adequate description of the cohort? Yes 5) Validated method for ascertaining exposure? Partial; classification of warfarin use/non-use within a given resident-month not explicated 6) Validated method for ascertaining clinical outcomes? Yes 7) Outcome assessment blind to exposure? Yes 8) Adequate follow-up period? Yes 9) Completeness of follow-up? Yes 10) Analysis controls for confounding? No; Did not analyze prognostic factors associated with adverse warfarin-related events other than warfarin exposure 11) Analytic methods appropriate? Partial; association of resident characteristics or INR values with risk of adverse warfarin-related event not assessed *Funding*: AHRQ

Gurwitz et al. (1997) [8]	To determine the prevalence of AF in the institutionalized elderly population and the proportion receiving warfarin; to identify clinical and functional characteristics of institutionalized elderly persons with AF that are associated with the use of warfarin; access quality of warfarin prescribing and monitoring	*Design*: retrospective cohort study *Data source*: medical record review of residents with ≥ 2 weeks of warfarin therapy during the 12-month period preceding the date of medical record abstraction	*Population*: 6437 residents of LTC facilities *Setting*: 30 LTC facilities (6437 total number of beds) located in New England, Quebec, and Ontario (US and Canada) *Time period*: Jul 1993-Aug 1995	An electrocardiogram indicating AF was present in the records of 7.5% of 5500 LTC residents; 32% of such patients were being treated with warfarin. In multivariate analysis, only a history of stroke (OR = 1.87; 95% CI = 1.20-2.91) was found to be positively associated with the use of warfarin in this setting. Patients with a diagnosis of dementia (OR = 0.59; 95% CI = 0.38-0.90) and those aged _85 years (OR = 0.46; 95% CI = 0.22-0.94) were less likely to receive warfarin therapy. Warfarin was commonly prescribed to patients with a history of bleeding (28.5%), substantial co-morbidity (30.8% major) and functional impairment (25.4% severe), a history of falls (28.5%), or concomitant potentiating drug therapy (17.7%)	*Quality assessment for observational studies:* 1) Unbiased selection of the cohort? Yes 2) Selection minimizes baseline differences in prognostic factors? Yes 3) Sample size calculated/5% difference? Yes 4) Adequate description of the cohort? Yes 5) Validated method for ascertaining exposure? Yes 6) Validated method for ascertaining clinical outcomes? Yes 7) Outcome assessment blind to exposure? Yes 8) Adequate follow-up period? Yes (cross-sectional) 9) Completeness of follow-up? Yes 10) Analysis controls for confounding? Yes 11) Analytic methods appropriate? Yes *Funding*: DuPont Pharma, the National Institute on Aging, Medical Research Council of the Province of Quebec

Hughes et al. (2004) [18]	To identify factors relating to initiation and discontinuation of secondary stroke prevention agents (warfarin and antiplatelets) among stroke survivors in nursing homes.	*Design*: retrospective cohort study *Data source*: MDS for patient characteristics matched to the OSCAR system for facility characteristics	*Population*: 16,579 stroke survivors; 9547 were not receiving any secondary stroke prevention treatment at admission; 6244 were receiving therapy *Setting*: nursing homes in 6 states (US) *Time period*: 1992-1996: each resident was followed ≥ 6 months	In all, 12% initiated drug therapy (warfarin or antiplatelet); 30.3% discontinued. Conditions known to increase the risk of recurrent stroke (e.g. AF) were predictive of initiation. Factors inversely related to initiation of therapy included advanced age, severe cognitive impairment, and being dependent in ADLs. Co-morbid conditions were inversely related to discontinuation of treatment, whereas advanced age and severe cognitive impairment increased likelihood of discontinuation. Black residents (OR = 0.62; 95% CI = 0.49-0.78) were less likely than non- Hispanic white residents to initiate therapy. Asian/Pacific Islanders (OR = 0.44; 95% CI = 0.23-0.83) were less likely than non-Hispanic white residents to discontinue therapy	*Quality assessment: for observational studies:* 1) Unbiased selection of the cohort? Yes 2) Selection minimizes baseline differences in prognostic factors? Yes 3) Sample size calculated/5% difference? Yes 4) Adequate description of the cohort? Yes 5) Validated method for ascertaining exposure? Yes 6) Validated method for ascertaining clinical outcomes? Yes 7) Outcome assessment blind to exposure? Yes 8) Adequate follow-up period? Partial; range of follow-up for observing initiation/discontinuation events was 6-13 months 9) Completeness of follow-up? Yes 10) Analysis controls for confounding? Partial; length of follow-up not treated as a covariate in adjusted logistic regression models 11) Analytic methods appropriate? Yes *Funding*: National Institute on Aging

Lackner et al. (1995) [9]	To assess warfarin use and monitoring in nursing home patients with NVAF, according to American College of Chest Physicians Consensus Conference guidelines	*Design*: retrospective, cross-sectional study *Data source*: medical record review and attending physician response to written communication from the nursing home's medical director and consultant pharmacist	*Population*: 902 patients aged ≥ 60 years, from whom 69 with a diagnosis of NVAF and 16 with VAF (control group) were identified *Setting*: 5 nursing homes in Minneapolis-St Paul, Minn (US) *Time period*: 1-month period (June 1993)	NVAF was documented in 7.6% and VAF in 1.8% of the patients. Only 17% of patients with NVAF were receiving warfarin, compared to 31% of patients with VAF. 58% of patients with NVAF and without a conventional contraindication to warfarin had ≥ 1 risk factor for thromboembolism in addition to AF and advanced age, yet only 20% used warfarin	*Quality assessment for observational studies:* 1) Unbiased selection of the cohort? Yes 2) Selection minimizes baseline differences in prognostic factors? Yes 3) Sample size calculated/5% difference? No; low power 4) Adequate description of the cohort? Yes 5) Validated method for ascertaining exposure? Yes 6) Validated method for ascertaining clinical outcomes? Yes 7) Outcome assessment blind to exposure? Yes 8) Adequate follow-up period? Yes (cross-sectional) 9) Completeness of follow-up? Yes 10) Analysis controls for confounding? Partial; evaluated univariate association of stroke risk factors and warfarin contraindications with warfarin use 11) Analytic methods appropriate? Partial; CIs not included in any findings *Funding*: Dupont Pharmaceuticals

Lapane et al. (2006) [19]	To evaluate the impact of the implementation of the Medicare PPS on pharmacologic secondary ischemic stroke prevention (standing orders for antiplatelets or warfarin) in nursing homes	*Design*: retrospective cohort study *Data source*: the SAGE database (including MDS data and all drugs taken 7 days preceding MDS assessment)	*Population*: residents who were hospitalized with an ischemic stroke within 6 months (1997, n = 5008; 2000, n = 5243) of living in nursing facilities *Setting*: nursing facilities in Kansas, Maine, Mississippi, or Ohio (1997: n = 1226; 2000: n = 1092) (US) *Time period*: Pre- PPS period = 1997; post-PPS period = 2000	The unadjusted proportion of use of pharmacologic agents for the secondary prevention of stroke was similar for warfarin in both time periods (1997: 22.9%; 2000: 22.4%) and increased for antiplatelets (1997:40.8%; 2000: 47.7%), as a result of the introduction of clopidogrel. Among residents with conditions indicating the use of warfarin, after adjusting for resident and facility characteristics, the likelihood of use of antiplatelets increased in the post-PPS era (adjusted OR = 1.26; 95% CI = 1.15-1.38); the likelihood of use of the use of warfarin did not change (adjusted OR = 0.99; 95% CI = 0.86-1.14)	*Quality assessment for observational studies:* 1) Unbiased selection of the cohort? Yes 2) Selection minimizes baseline differences in prognostic factors? Partial; design does control for effects of history other than implementation of PPS between pre- and post-PPS period (e.g. issuance of prescribing guidelines) 3) Sample size calculated/5% difference? Yes 4) Adequate description of the cohort? Yes 5) Validated method for ascertaining exposure? Yes 6) Validated method for ascertaining clinical outcomes? Yes 7) Outcome assessment blind to exposure? Yes 8) Adequate follow-up period? Partial; evaluated only 1 pre- and post-PPS year; cross-sectional 9) Completeness of follow-up? Yes 10) Analysis controls for confounding? Yes 11) Analytic methods appropriate? Yes *Funding*: supported by a National Primary Care Career Scientist Award from the Research and Development Office, Northern Ireland

Lau et al. (2004) [10]	To identify patterns and predictors of antithrombotic use and to evaluate the appropriateness of antithrombotic therapy for stroke prophylaxis in institutionalized elderly patients with AF	*Design*: retrospective cohort study *Data source*: Administrative databases and medical records	*Population*: 265 LTC residents, aged ≥ 65 and older, with AF *Setting*: 17 LTC institutions in Edmonton, Alberta (Canada) *Time period*: Nov 2001 - Feb 2002	Warfarin was prescribed for 49% of patients, aspirin for 22%, both for 8%, and neither for 20%. Nearly all patients (97%) were considered to be at high risk for stroke, with age being the predominant risk factor (88% ≥ 75 years), whereas about half (54%) were considered to be at low risk for bleeding. Multivariate analyses found no associations between individual risk factors for bleeding and anticoagulation treatment, with the exception of recent surgery (OR = 0.59; 95% CI = 0.37-0.94). Overall, 54.8% of patients received appropriate antithrombotic therapy congruent with stroke and bleeding. Of patients who were optimal candidates for anticoagulation, 60% received appropriate therapy (warfarin with or without aspirin)	*Quality assessment for observational studies:* 1) Unbiased selection of the cohort? Yes 2) Selection minimizes baseline differences in prognostic factors? Yes 3) Sample size calculated/5% difference? Yes 4) Adequate description of the cohort? Yes 5) Validated method for ascertaining exposure? Partial; unlike stroke risk, categorization by bleeding risk not based on validated algorithm or consensus guideline 6) Validated method for ascertaining clinical outcomes? Yes 7) Outcome assessment blind to exposure? Yes 8) Adequate follow-up period? Yes; cross-sectional 9) Completeness of follow-up? Yes 10) Analysis controls for confounding? Yes 11) Analytic methods appropriate? Yes *Funding*: not specified

McCormick et al. (2001) [11]	To assess: (1) the prevalence of AF and the percentage of AF patients who receive therapy with warfarin or aspirin, (2) the relationship between the presence of known risk factors for stroke and bleeding among persons with AF and their receipt of warfarin, and (3) the quality of warfarin prescribing and monitoring in nursing home residents with AF	*Design*: retrospective cohort study *Data source*: Medical record review	*Population*: 2587 LTC residents *Setting*: 21 LTC facilities in Connecticut (US) *Time period*: 1997-1998	AF was present in 17% of LTC residents, risk factors for stroke in 93% of AF residents, and for bleeding in 80% of AF residents. Overall, 42% of AF patients were receiving warfarin. However, of 83 ideal candidates, only 53% were receiving this therapy. The odds of receiving warfarin in the study sample decreased with increasing number of risk factors for bleeding (adjusted OR for > 1 bleeding risk factor compared to none: 0.51; CI, 0.29-0.94) and increased (non-significant trend) with increasing number of stroke risk factors	*Quality assessment for observational studies:* 1) Unbiased selection of the cohort? Yes 2) Selection minimizes baseline differences in prognostic factors? Yes 3) Sample size calculated/5% difference? Yes 4) Adequate description of the cohort? Yes 5) Validated method for ascertaining exposure? No; stroke risk and bleeding risk classification not adequately described (i.e. no reference to validated algorithm or consensus guideline) 6) Validated method for ascertaining clinical outcomes? Yes 7) Outcome assessment blind to exposure? Yes 8) Adequate follow-up period? Yes; cross-sectional 9) Completeness of follow-up? Yes 10) Analysis controls for confounding? Partial; a small list of potential confounders was included in the logistic regression model 11) Analytic methods appropriate? Yes *Funding*: Health Care Financing Administration, Department of Health and Human Services

Quilliam et al. (2001) [12]	To explore characteristics of nursing home residents who are stroke survivors and factors associated with secondary prevention of stroke in nursing homes	*Design*: Retrospective cross-sectional study *Data source*: MDS	*Population*: 53,829 (20.4%) residents aged > 65 years with a diagnosis of stroke (stroke type unknown) *Setting*: all nursing home residents in 5 states (US) *Time period*: 1992-1995	67% of stroke survivors and > 50% of those hospitalized with stroke over the previous 6 months were not receiving drug therapy for stroke prevention. Among those treated, most received aspirin alone (16%) or warfarin alone (10%). Independent predictors of drug treatment included co-morbid conditions (e.g. hypertension, AF, depression, Alzheimer's disease, dementia, history of GI bleeding, and peptic ulcer disease). Those aged ≥ 85 years were less likely to be treated than those aged 65-74 years (OR = 0.86; 95% CI = 0.82-0.91); black residents were less likely to be treated than whites (OR = 0.80; 95% CI = 0.75-0.85); and those with severe cognitive (OR = 0.63; 95% CI = 0.60-0.67) or physical impairment (OR = 0.69; 95% CI = 0.64-0.75) were also less likely to receive drug treatment	*Quality assessment for observational studies:* 1) Unbiased selection of the cohort? Yes 2) Selection minimizes baseline differences in prognostic factors? Yes 3) Sample size calculated/5% difference? Yes 4) Adequate description of the cohort? Yes 5) Validated method for ascertaining exposure? Partial; only a limited set of bleeding risk factors were considered in the logistic regression model 6) Validated method for ascertaining clinical outcomes? Yes 7) Outcome assessment blind to exposure? Yes 8) Adequate follow-up period? Yes; cross-sectional 9) Completeness of follow-up? Yes 10) Analysis controls for confounding? Yes 11) Analytic methods appropriate? Yes *Funding*: National Institute on Aging, AHRQ

Sloane et al. (2004) [13]	To determine the prevalence and predictors of non-prescribing of selected medications for 4 common geriatric conditions (including aspirin or anticoagulants for persons with a history of stroke) whose value in decreasing morbidity has been established in clinical trials	*Design*: Cross-sectional study *Data source*: patient characteristics and diagnoses were based on medical record reviews and in-person patient assessments; data on facility characteristics were obtained by interviewing facility administrators	*Population*: 2014 residents aged ≥ 65 years *Setting*: a stratified random sample of 193 residential care/assisted living facilities in Florida, Maryland, New Jersey, and North Carolina (US) *Time period*: Oct 1997 - Nov 1998	Of 435 patients with prior stroke (stroke type not specified) 14.4% had a contraindication for aspirin use and 0% had a contraindication for warfarin use. 37.5% were not receiving an anticoagulant or antiplatelet agent. Neither bivariate nor multivariate analysis showed an association between non-prescribing and resident characteristics. Some facility characteristics were associated with non-prescribing in bivariate analysis (traditional vs small facility [OR = 0.55; *P *< 0.05], new model vs small facility [OR = 0.47; *P *< 0.01], presence of an RN/LPN [OR = 0.58; *P *< 0.05]). However, in the multivariate analysis no facility characteristics were significantly associated with Non-prescribing	*Quality assessment for observational studies:* 1) Unbiased selection of the cohort? Yes 2) Selection minimizes baseline differences in prognostic factors? Yes 3) Sample size calculated/5% difference? Yes 4) Adequate description of the cohort? Yes 5) Validated method for ascertaining exposure? No; contraindication to warfarin use not evaluated; contraindication to aspirin use limited to peptic ulcer disease 6) Validated method for ascertaining clinical outcomes? Yes 7) Outcome assessment blind to exposure? Yes 8) Adequate follow-up period? Yes; cross-sectional 9) Completeness of follow-up? Yes 10) Analysis controls for confounding? Partial; many covariates in multiple drug therapy study have little relevance to warfarin or antiplatelet use 11) Analytic methods appropriate? Yes *Funding*: National Institute on Aging

**Part B: Medication management interventions**					

Crotty et al. (2004) [20]	To assess whether pharmacist outreach visits would improve the implementation of evidence-based clinical practice in the area of falls reduction and stroke prevention in a residential care setting	*Design*: randomized control trial *Data source*: pre- and post-intervention case note audits	*Population*: 452 residential care staff was surveyed; 121 physicians were involved, with 61 receiving outreach visits. Pre- and post-intervention data were available for 715 LTC residents *Setting*: 10 nursing homes and 10 hostels (low-level facilities) in South Australia *Time period*: 7-month follow-up period (dates not specified)	No statistically significant difference between groups for numbers of patients at risk of stroke on aspirin at follow-up. Percent of residents with AF recorded on warfarin was similar between groups: 22.6% (pre) and 17.1% (post) in the control group, and 8.6% (pre) and 16.7% (post) in the intervention group (RR = 0.92; 95% CI = 0.23-3.95)	*Quality assessment for observational studies:* 1) Baseline comparability? Yes 2) Valid AD/cognitive outcomes assessment? Yes 3) Subjects/providers blind? Cannot be determined 4) Outcome assessors blind? Yes 5) Incomplete data adequately addressed? Yes 6) Differential dropout rate < 10%? Yes 7) Overall dropout rate < 30%? Yes; was 22.5% but as high as 37% in some cluster facilities 8) Conflict of interest reported and insignificant? Yes 9) Randomization adequate? Yes 10) Allocation concealment adequate? Yes *Funding*: National Health & Medical Research Council Evidence Based Clinical Practice Research Program

Horning et al. (2007) [21]	To evaluate clinical practice guideline adherence (including antiplatelet and anticoagulation therapy for secondary stroke in prevention) in patients LTC facilities who received pharmacist-directed DSM compared with patients in other LTC facilities who received traditional DRR	*Design*: retrospective cohort study *Data source*: chart review	*Population*: for the secondary stroke prevention subgroup, 18 stroke patients who received DSM services and 86 stroke patients who received DRR services *Setting*: DSM services (intervention) in 2 LTC facilities and DRR services (control) in 4 LTC facilities (US) *Time period*: Nov 2005	For patients with prior stroke, more DSM vs DDR patients received aspirin, clopidogrel or warfarin or were recognized with a contraindication (unadjusted, 88.9% vs 69.8%; *P *= 0.096; adjusted OR = 5.380; 95% CI = 0.975- 29.684)	*Quality assessment for observational studies:* 1) Unbiased selection of the cohort? No; control group was determined retrospectively 2) Selection minimizes baseline differences in prognostic factors? Partial; control group was determined by authors to be representative mix of local usual pharmacist consultant services 3) Sample size calculated/5% difference? No; intervention group may have lacked power due to low n = 107 residents 4) Adequate description of the cohort? Yes 5) Validated method for ascertaining exposure? Partial; description of DSM intervention for each of seven diseases evaluated was limited 6) Validated method for ascertaining clinical outcomes? Partial; description of guideline adherence scoring limited; only cite consensual guidelines 7) Outcome assessment blind to exposure? No 8) Adequate follow-up period? Yes 9) Completeness of follow-up? Yes 10) Analysis controls for confounding? No; did not adjust for institutional characteristics in logistic regression models 11) Analytic methods appropriate? No; limited covariate set unchanged among seven disease logistic regression models; stroke and bleeding risk not adequately modeled; no multiplicity adjustment *Funding*: No outside funding.

Papaioannou et al. (2010) [24]	To evaluate the MEDeINR system (an electronic decision support system based on a validated algorithm for warfarin dosing) by examining the impact on INR control, testing frequency, and experiences of staff in using the system	*Design*: retrospective/prospective cohort study (pre-post implementation design) *Data source*: pre-implementation: retrospective chart audit; post-implementation: central computer database	*Population*: 128 residents (without prosthetic valve) who were taking warfarin *Setting*: 6 LTC homes in Ontario (Canada) *Time period*: 6 months, 3 months prior to MEDeINR implementation and 3 months post-implementation (dates not specified)	128 (10%) of all residents (excluding those with a prosthetic valve) were taking warfarin in 6 LTC homes. The primary indications for taking warfarin were: AF (74%), DVT (20%), and PE (6%)	*Quality assessment for observational studies:* 1) Unbiased selection of the cohort? No; potential survivor bias since residents who discontinued warfarin prior to intervention due to poor INR control would not have been eligible for study 2) Selection minimizes baseline differences in prognostic factors? Partial; pre- and post-intervention without control does not adjust for biases such as history and maturation 3) Sample size calculated/5% difference? Yes 4) Adequate description of the cohort? Yes 5) Validated method for ascertaining exposure? Yes 6) Validated method for ascertaining clinical outcomes? Yes 7) Outcome assessment blind to exposure? No 8) Adequate follow-up period? Partial; some residents had < 3 months of follow-up 9) Completeness of follow-up? No; differential follow-up in pre- and post- periods not evaluated 10) Analysis controls for confounding? Partial; no covariates modeled since subjects served as own controls; assumes no time-varying relevant covariates 11) Analytic methods appropriate? Partial; sensitivity analysis to test survivor bias not performed *Funding*: Canadian Institute of Health Research

ADL, activities of daily living; AF, atrial fibrillation; AHRQ, Agency for Healthcare Research and Quality; CI, confidence interval; DSM, disease state management; DRR, drug regimen review; DVT, deep vein thrombosis; GI, gastrointestinal; INR, international normalized ratio; LTC, long-term care; LPN, licensed practical nurse; MDS, Minimum Data Set; NVAF, nonvalvular atrial fibrillation; OAC, oral anticoagulation; OR, odds ratio; OSCAR, Online Survey Certification and Automated Record; PE, pulmonary embolism; PPS, prospective payment system; RN, registered nurse; RR, relative risk; SAGE, Systematic Assessment of Geriatric drug use via Epidemiology; VAF, valvular atrial fibrillation

#### Primary indications for warfarin therapy

AF was the primary indication for warfarin therapy in two studies (n = 618), accounting for 74% [24] and 58% [25] of residents receiving warfarin, respectively. Stroke prevention without AF accounted for 12% [25], while DVT accounted for 20% [24], PE for 6% [24], and either DVT or PE for 26% [25] of residents receiving warfarin.

#### Rate of warfarin use

Eight studies (n = 22,573) [7-13,19] reported rates of warfarin use across all residents having a condition for which warfarin was indicated. These only included residents having AF or previous stroke; no other indications were described. Four of these studies (n = 2396) [7,10,12,13] also reported the rates of use for warfarin combined with antiplatelet therapy. Rates for warfarin use alone ranged from 17% [9] to 57% [10], while the rate of either warfarin or antiplatelet use ranged from 62% [13] to 80% [10].

#### Rate of warfarin use among AF patients

Of the eight studies above, five (n = 10,308) [7-11] reported rates of warfarin use among LTC residents with AF that ranged from 17% [9] to 57%[10]. Abdel-Latif and colleagues [7] found that 46% of 117 residents with AF in six LTC facilities had been prescribed warfarin; 79% received either warfarin, aspirin, or clopidogrel. In a study of 265 LTC residents with AF in Canada from 2001-2002, Lau et al.[10] found that warfarin was prescribed for 57%; among residents who were considered optimal candidates (high risk of stroke and low risk of bleeding according to the criteria used in this study [10]), the warfarin prescribing rate was 60%. Using 1993 data, Lackner et al. [9], in a study of five LTC facilities, found that only 17% of patients with non-valvular AF received warfarin; among residents with AF with ≥ 1 additional risk factor for stroke (besides AF) and no contraindication to warfarin use, only 20% received warfarin [9]. Gurwitz et al. [8], using data from 1993 to 1995, found rates of warfarin use of 32% in 413 residents with AF. Finally, in a study of 21 LTC facilities from 1997 to 1998, McCormick et al. [11] reported that 42% of residents with AF received warfarin, and only 53% of ideal AF candidates for warfarin therapy (those having no bleeding risk factors) received it.

#### Rate of warfarin use among stroke patients

Three studies measured the use of warfarin in LTC facilities among stroke survivors (Table 1 Part A) [12,13,19]. Lapane et al. [19] evaluated, among stroke survivors, whether introduction of a prospective payment system (PPS) that required nursing homes to bear the cost for warfarin monitoring had shifted utilization from warfarin to antiplatelet agents. Comparing data from 1997 and 2000, Lapane et al. [19] found that the use of warfarin for the secondary prevention of ischemic stroke did not change significantly following the introduction of the PPS, from 22% in 1997 to 23% in 2000, while antiplatelet use did increase from 41% to 48% over the same period (likely due to the introduction of clopidogrel). Sloane et al. [13] examined 1997-1998 data from residential care/assisted living facilities in the United States and found that 38% of the 435 residents with a history of stroke (type not specified) received neither warfarin nor an antiplatelet agent. Quilliam et al. [12] analyzed records of 53,829 survivors of stroke (either ischemic or hemorrhagic) in all nursing homes in five states from 1992 to 1995 (SAGE database) and found that 67% were not receiving warfarin or any antiplatelet medication for stroke prevention; among residents recently hospitalized for ischemic stroke, 52% did not receive either of these agents.

### Association of Resident and/or Facility Characteristics with Warfarin Prescribing

Eight studies [7,8,10-13,17,18] (a subset of the studies shown in Table 1; n = 100,879), explored the relationship between warfarin or combined warfarin/antiplatelet usage and resident or facility characteristics. All studies used multivariate models to adjust for potential confounders. The direction (negative, positive, none noted [i.e. not significant]) and strength (reported odds ratio [OR]) of reported associations within these multivariate models are summarized in Table 2.

**Table 2:** Association of factors with warfarin prescribing

Category	Factor	Direction of association (at 95% confidence) 0 = none + = positive - = negative	Association (multivariate adjusted) (OR, 95% CI)	Endpoint	Study Condition	Study
Admission	Admitted from hospital	+	OR = 1.16 (1.02-1.31)	*discontinue warfarin or antiplatelets*	Previous stroke	Hughes et al. (2004) [18]
		
		0	OR = 1.12 (0.97-1.29)	initiate warfarin or antiplatelets	Previous stroke	Hughes et al. (2004) [18]

Age	65-74	0	OR = 0.98 (0.61-1.57)	use of warfarin	AF	Lau et al. (2004) [10]
	
	75-84	0	OR = 0.98 (0.61-1.58)	use of warfarin	AF	Lau et al. (2004) [10]
		
		0	OR = 1.13 (0.98-1.31)	*discontinue warfarin or antiplatelets*	Previous stroke	Hughes et al. (2004) [18]
		
		0	OR = 0.99 (0.94-1.04)	use of warfarin or antiplatelets	Previous stroke	Quilliam et al. (2001) [12]
		
		0	not reported	use of warfarin	AF	Gurwitz et al. (1997) [8]
		
		0	OR = 1.01 (0.86-1.19)	initiate warfarin or antiplatelets	Previous stroke	Hughes et al. (2004) [18]
	
	≥ 85	0	OR = 1.13 (0.70-1.82)	use of warfarin	AF	Lau et al. (2004) [10]
		
		0	OR = 1.07 (CI not reported)	use of warfarin or antiplatelets	Previous stroke	Sloane et al. (2004) [13]
		
		+	OR = 1.23 (1.05-1.43)	*discontinue warfarin or antiplatelets*	Previous stroke	Hughes et al. (2004) [18]
		
		-	OR = 0.46 (0.22-0.94)	use of warfarin	AF	Gurwitz et al. (1997) [8]
		
		-	OR = 0.86 (0.82-0.91)	use of warfarin or antiplatelets	Previous stroke	Quilliam et al. (2001) [12]
		
		0	OR = 0.86 (0.72-1.04)	initiate warfarin or antiplatelets	Previous stroke	Hughes et al. (2004) [18]

Bleeding risk	1 risk factor	0	OR = 0.75 (0.41-1.36)	use of warfarin	AF	McCormick et al. (2001) [11]
		
	≥ 2 risk factors	-	OR = 0.51 (0.29-0.94)	use of warfarin	AF	McCormick et al. (2001) [11]
		
	High risk	0	OR = 0.82 (0.52-1.30)	use of warfarin	AF	Lau et al. (2004) [10]

Cognitive impairment	Moderate	-	OR = 0.93 (0.88-0.97)	use of warfarin or antiplatelets	Previous stroke	Quilliam et al. (2001) [12]
		
		0	OR = 0.93 (0.81-1.08)	initiate warfarin or antiplatelets	Previous stroke	Hughes et al. (2004) [18]
		
		0	OR = 0.98 (0.86-1.12)	*discontinue warfarin or antiplatelets*	Previous stroke	Hughes et al. (2004) [18]
	
	Severe	0	OR = 1.19 (0.99-1.44)	*discontinue warfarin or antiplatelets*	Previous stroke	Hughes et al. (2004) [18]
		
		-	OR = 0.64 (0.52-0.80)	initiate warfarin or antiplatelets	Previous stroke	Hughes et al. (2004) [18]
		
		-	OR = 0.63 (0.60-0.67)	use of warfarin or antiplatelets	Previous stroke	Quilliam et al. (2001) [12]
		
		0	OR = 1.02 (CI not reported)	use of warfarin or antiplatelets	Previous stroke	Sloane et al. (2004) [13]

Conditions	Active malignancy	0	OR = 0.93 (0.57-1.51)	use of warfarin	AF	Lau et al. (2004)[10]
	
	Alzheimer's disease	-	OR = 0.77 (0.70-0.85)	use of warfarin or antiplatelets	Previous stroke	Quilliam et al. (2001) [12]
	
	Anemia	0	OR = 0.87 (0.55-1.39)	use of warfarin	AF	Lau et al. (2004) [10]
	
	Aneurysms	0	OR = 0.88 (0.55-1.40)	use of warfarin	AF	Lau et al. (2004) [10]
	
	Atrial fibrillation	-	OR = 0.73 (0.64-0.83)	*discontinue warfarin or antiplatelets*	Previous stroke	Hughes et al. (2004) [18]
		
		+	OR = 1.76 (1.50-2.06)	initiate warfarin or antiplatelets	Previous stroke	Hughes et al. (2004) [18]
		
		+	OR = 2.04 (1.95-2.14)	use of warfarin or antiplatelets	Previous stroke	Quilliam et al. (2001) [12]
	
	Congestive heart failure	0	OR = 1.13 (0.98-1.30)	*discontinue warfarin or antiplatelets*	Previous stroke	Hughes et al. (2004) [18]
		
		0	OR = 1.04 (0.65-1.65)	use of warfarin	AF	Lau et al. (2004) [10]
		
		0	not reported	use of warfarin	AF	Abdel-Latif et al. (2005) [7]
		
		0	OR = 1.02 (0.87-1.20)	initiate warfarin or antiplatelets	Previous stroke	Hughes et al. (2004) [18]
	
	Coronary artery disease	+	OR = 1.06 (1.02-1.11)	use of warfarin or antiplatelets	Previous stroke	Quilliam et al. (2001) [12]
		
		0	OR = 0.99 (0.62-1.58)	use of warfarin	AF	Lau et al. (2004) [10]
	
	Dementia	-	OR = 0.84 (0.80-0.88)	use of warfarin or antiplatelets	Previous stroke	Quilliam et al. (2001) [12]
		
		-	OR = 0.59 (0.38-0.90)	use of warfarin	AF	Gurwitz et al. (1997) [8]
	
	Depression	+	OR = 1.22 (1.02-1.46)	initiate warfarin or antiplatelets	Previous stroke	Hughes et al. (2004) [18]
		
		+	OR = 1.11 (1.05-1.18)	use of warfarin or antiplatelets	Previous stroke	Quilliam et al. (2001) [12]
		
		0	OR = 1.08 (0.93-1.24)	*discontinue warfarin or antiplatelets*	Previous stroke	Hughes et al. (2004) [18]
	
	Diabetes mellitus	0	not reported	use of warfarin	AF	Abdel-Latif et al. (2005) [7]
		
		0	OR = 1.17 (0.73-1.86)	use of warfarin	AF	Lau et al. (2004) [10]
	
	Hypertension	-	OR = 0.87 (0.78-0.97)	*discontinue warfarin or antiplatelets*	Previous stroke	Hughes et al. (2004) [18]
		
		+	OR = 1.23 (1.09-1.39)	initiate warfarin or antiplatelets	Previous stroke	Hughes et al. (2004) [18]
		
		0	OR = 1.10 (0.69-1.75)	use of warfarin	AF	Lau et al. (2004) [10]
		
		0	not reported	use of warfarin	AF	Abdel-Latif et al. (2005) [7]
		
		+	OR = 1.27 (1.22-1.32)	use of warfarin or antiplatelets	Previous stroke	Quilliam et al. (2001) [12]
	
	Left ventricular dysfunction	0	OR = 0.83 (0.63-1.09)	initiate warfarin or antiplatelets	Previous stroke	Hughes et al. (2004) [18]
		
		0	OR = 1.07 (0.88-1.30)	*discontinue warfarin or antiplatelets*	Previous stroke	Hughes et al. (2004) [18]
	
	Liver disease	0	OR = 1.53 (0.95-2.49)	use of warfarin	AF	Lau et al. (2004) [10]
	
	Major comorbidity burden	0	not reported	use of warfarin	AF	Gurwitz et al. (1997) [8]
	
	Moderate comorbidity burden	0	not reported	use of warfarin	AF	Gurwitz et al. (1997) [8]
	
	Multiple conditions (4 or more)	0	OR = 0.84 (CI not reported)	use of warfarin or antiplatelets	Previous stroke	Sloane et al. (2004) [13]
	
	Peptic ulcer disease	0	OR = 0.90 (0.57-1.43)	use of warfarin	AF	Lau et al. (2004) [10]
		
		-	OR = 0.64 (0.58-0.71)	use of warfarin or antiplatelets	Previous stroke	Quilliam et al. (2001) [12]
	
	Peripheral vascular disease	+	OR = 1.13 (1.05-1.20)	use of warfarin or antiplatelets	Previous stroke	Quilliam et al. (2001) [12]
	
	Previous bleeding	0	not reported	use of warfarin	AF	Gurwitz et al. (1997) [8]
	
	Previous falls	0	not reported	use of warfarin	AF	Gurwitz et al. (1997) [8]
	
	Previous GI bleeding	-	OR = 0.57 (0.52-0.62)	use of warfarin or antiplatelets	Previous stroke	Quilliam et al. (2001) [12]
		
		-	OR = 0.18 (0.03-0.91)	use of warfarin	AF	Abdel-Latif et al. (2005) [7]
	
	Previous major bleeding	0	OR = 0.73 (0.46-1.15)	use of warfarin	AF	Lau et al. (2004) [10]
	
	Previous stroke	+	OR = 4.93 (2.11-11.49)	use of warfarin	AF	Abdel-Latif et al. (2005) [7]
		
		+	OR = 1.87 (1.20-2.91)	use of warfarin	AF	Gurwitz et al. (1997) [8]
	
	Previous stroke or TIA	0	OR = 1.24 (0.78-1.97)	use of warfarin	AF	Lau et al. (2004) [10]
	
	Previous systemic embolus	0	OR = 1.46 (0.92-2.34)	use of warfarin	AF	Lau et al. (2004) [10]
	
	Recent surgery	-	OR = 0.59 (0.37-0.94)	use of warfarin	AF	Lau et al. (2004) [10]
	
	Renal insufficiency	0	OR = 0.91 (0.57-1.45)	use of warfarin	AF	Lau et al. (2004) [10]
	
	Rheumatic mitral valvular	0	OR = 0.80 (0.50-1.28)	use of warfarin	AF	Lau et al. (2004) [10]
	
	Seizure disorder	0	OR = 1.05 (0.66-1.67)	use of warfarin	AF	Lau et al. (2004) [10]
	
	Transient ischemic attack	+	OR = 1.34 (1.09-1.64)	initiate warfarin or antiplatelets	Previous stroke	Hughes et al. (2004) [18]
		
		0	OR = 0.99 (0.84-1.17)	*discontinue warfarin or antiplatelets*	Previous stroke	Hughes et al. (2004) [18]

Drug Interaction	Uses meds that increase bleeding risk	0	not reported	use of warfarin	AF	Gurwitz et al. (1997) [8]
		
		0	OR = 1.26 (0.80-1.98)	use of warfarin	AF	Lau et al. (2004) [10]

Duration of AF	12-24 months	0	not reported	use of warfarin	AF	Gurwitz et al. (1997) [8]
	
	> 24 months	0	not reported	use of warfarin	AF	Gurwitz et al. (1997) [8]
	
	Onset of AF after admission	0	not reported	use of warfarin	AF	Gurwitz et al. (1997) [8]

Facility	Alzheimer's unit	0	OR = 0.78 (0.57-1.05)	*discontinue warfarin or antiplatelets*	Previous stroke	Hughes et al. (2004) [18]
		
		0	OR = 1.14 (0.80-1.62)	initiate warfarin or antiplatelets	Previous stroke	Hughes et al. (2004) [18]
	
	Hospital based	0	OR = 0.96 (0.72-1.29)	initiate warfarin or antiplatelets	Previous stroke	Hughes et al. (2004) [18]
		
		0	OR = 0.96 (0.75-1.23)	*discontinue warfarin or antiplatelets*	Previous stroke	Hughes et al. (2004) [18]
	
	Location rural	0	OR = 0.89 (CI not reported)	use of warfarin or antiplatelets	Previous stroke	Sloane et al. (2004) [13]
	
	Location urban	+	OR = 1.38 (1.16-1.65)	*discontinue warfarin or antiplatelets*	Previous stroke	Hughes et al. (2004) [18]
		
		0	OR = 1.06 (0.87-1.3)	initiate warfarin or antiplatelets	Previous stroke	Hughes et al. (2004) [18]
	
	Large new model facility (vs small)	0	OR = 0.61 (CI not reported)	use of warfarin or antiplatelets	Previous stroke	Sloane et al. (2004) [13]
	
	Non-white > 10% (vs > 0% to < 5%)	0	OR = 1.09 (0.91-1.32)	initiate warfarin or antiplatelets	Previous stroke	Hughes et al. (2004) [18]
		
		+	OR = 1.22 (1.03-1.43)	*discontinue warfarin or antiplatelets*	Previous stroke	Hughes et al. (2004) [18]
	
	Non-white > 5% to < 10% (vs > 0% to < 5%)	0	OR = 1.00 (0.81-1.24)	initiate warfarin or antiplatelets	Previous stroke	Hughes et al. (2004) [18]
		
		0	OR = 0.95 (0.78-1.15)	*discontinue warfarin or antiplatelets*	Previous stroke	Hughes et al. (2004) [18]
	
	Non-white 0% (vs > 0% to < 5%)	-	OR = 0.74 (0.57-0.96)	initiate warfarin or antiplatelets	Previous stroke	Hughes et al. (2004) [18]
		
		0	OR = 1.17 (0.93-1.46)	*discontinue warfarin or antiplatelets*	Previous stroke	Hughes et al. (2004) [18]
	
	Ownership status for profit (vs non-profit)	0	OR = 0.90 (0.76-1.07)	initiate warfarin or antiplatelets	Previous stroke	Hughes et al. (2004) [18]
		
		0	OR = 0.90 (0.77-1.05)	*discontinue warfarin or antiplatelets*	Previous stroke	Hughes et al. (2004) [18]
	
	Ownership status government (vs non-profit)	0	OR = 0.86 (0.64-1.17)	initiate warfarin or antiplatelets	Previous stroke	Hughes et al. (2004) [18]
		
		0	OR = 0.99 (0.76-1.29)	*discontinue warfarin or antiplatelets*	Previous stroke	Hughes et al. (2004) [18]
	
	Part of a chain	+	OR = 1.20 (1.01-1.42)	initiate warfarin or antiplatelets	Previous stroke	Hughes et al. (2004) [18]
		
		-	OR = 0.85 (0.73-0.99)	*discontinue warfarin or antiplatelets*	Previous stroke	Hughes et al. (2004) [18]
	
	Payment source % Medicaid (per 10 unit increase)	0	OR = 0.98 (0.91-1.05)	*discontinue warfarin or antiplatelets*	Previous stroke	Hughes et al. (2004) [18]
		
		0	OR = 0.95 (0.88-1.03)	initiate warfarin or antiplatelets	Previous stroke	Hughes et al. (2004) [18]
	
	Payment source % other-pay (per 10 unit increase)	0	OR = 0.94 (0.87-1.01)	*discontinue warfarin or antiplatelets*	Previous stroke	Hughes et al. (2004) [18]
		
		0	OR = 0.97 (0.89-1.05)	initiate warfarin or antiplatelets	Previous stroke	Hughes et al. (2004) [18]
	
	Presence of a RN/LPN	0	OR = 0.74 (CI not reported)	use of warfarin or antiplatelets	Previous stroke	Sloane et al. (2004) [13]
	
	Size ≤ 80 (vs 81-199)	0	OR = 1.01 (0.81-1.26)	initiate warfarin or antiplatelets	Previous stroke	Hughes et al. (2004) [18]
		
		0	OR = 0.92 (0.77-1.11)	*discontinue warfarin or antiplatelets*	Previous stroke	Hughes et al. (2004) [18]
	
	Size ≥ 200 (vs 81 to 199)	0	OR = 1.17 (0.99-1.39)	*discontinue warfarin or antiplatelets*	Previous stroke	Hughes et al. (2004) [18]
		
		0	OR = 1.08 (0.90-1.30)	initiate warfarin or antiplatelets	Previous stroke	Hughes et al. (2004) [18]
	
	Special care unit	0	OR = 1.15 (0.83-1.59)	initiate warfarin or antiplatelets	Previous stroke	Hughes et al. (2004) [18]
		
		+	OR = 1.33 (1.02-1.73)	*discontinue warfarin or antiplatelets*	Previous stroke	Hughes et al. (2004) [18]
	
	Staff resources any full-time physicians	-	OR = 0.76 (0.63-0.92)	initiate warfarin or antiplatelets	Previous stroke	Hughes et al. (2004) [18]
		
		0	OR = 1.05 (0.89-1.24)	*discontinue warfarin or antiplatelets*	Previous stroke	Hughes et al. (2004) [18]
	
	Staff resources (contract)	0	OR = 1.04 (0.89-1.20)	initiate warfarin or antiplatelets	Previous stroke	Hughes et al. (2004) [18]
		
		0	OR = 1.02 (0.89-1.17)	*discontinue warfarin or antiplatelets*	Previous stroke	Hughes et al. (2004) [18]
	
	Staff resources (physician extenders)	+	OR = 1.21 (1.0-1.47)	*discontinue warfarin* *or antiplatelets*	Previous stroke	Hughes et al. (2004) [18]
		
		0	OR = 1.08 (0.87-1.34)	initiate warfarin or antiplatelets	Previous stroke	Hughes et al. (2004) [18]
	
	Traditional facility (vs small)	0	OR = 0.78 (CI not reported)	use of warfarin or antiplatelets	Previous stroke	Sloane et al. (2004) [13]
	
	Weekly physician visits	0	OR = 0.94 (CI notreported)	use of warfarin or antiplatelets	Previous stroke	Sloane et al. (2004) [13]

Gender	Female	0	not reported	use of warfarin	AF	Gurwitz et al. (1997) [8]
		
		-	OR = 0.94 (0.90-0.98)	use of warfarin orantiplatelets	Previous stroke	Quilliam et al. (2001) [12]
		
		0	OR = 0.99 (0.87-1.13)	initiate warfarin orantiplatelets	Previous stroke	Hughes et al. (2004) [18]
		
		0	OR = 1.00 (0.89-1.13)	*discontinue warfarinor antiplatelets*	Previous stroke	Hughes et al. (2004) [18]
		
		0	OR = 0.81 (CI not reported)	use of warfarin orantiplatelets	Previous stroke	Sloane et al. (2004) [13]

Physical Function	Substantial mobility	0	not reported	use of warfarin	AF	Gurwitz et al. (1997) [8]
	
	Mild impairment	0	not reported	use of warfarin	AF	Gurwitz et al. (1997) [8]
	
	Intermediate mobility	0	not reported	use of warfarin	AF	Gurwitz et al. (1997) [8]
	
	Moderate impairment	0	OR = 0.95 (0.75-1.20)	*discontinue warfarin or antiplatelets*	Previous stroke	Hughes et al. (2004) [18]
		
		0	OR = 0.90 (0.70-1.16)	initiate warfarin or antiplatelets	Previous stroke	Hughes et al. (2004) [18]
		
		0	not reported	use of warfarin	AF	Gurwitz et al. (1997) [8]
		
		0	OR = 1.03 (0.95-1.11)	use of warfarin or antiplatelets	Previous stroke	Quilliam et al. (2001) [12]
	
	Dependent	-	OR = 0.69 (0.64-0.75)	use of warfarin or antiplatelets	Previous stroke	Quilliam et al. (2001) [12]
		
		-	OR = 0.73 (0.56-0.96)	initiate warfarin or antiplatelets	Previous stroke	Hughes et al. (2004) [18]
		
		0	OR = 0.99 (0.79-1.25)	*discontinue warfarin or antiplatelets*	Previous stroke	Hughes et al. (2004) [18]
		
		0	OR = 1.21 (CI notreported)	use of warfarin or antiplatelets	Previous stroke	Sloane et al. (2004) [13]
	
	Severe impairment	0	not reported	use of warfarin	AF	Gurwitz et al. (1997) [8]

Race/ethnicity	American Indian	0	OR = 1.00 (0.70-1.43)	*discontinue warfarin or antiplatelets*	Previous stroke	Hughes et al. (2004) [18]
		
		0	Difference = -0.8(-8.9 to 7.3)	prevalence difference from non-Hispanic white for receiving warfarin or antiplatelets	Recent ischemic stroke	Christian et al. (2003) [17]
		
		0	OR = 1.47 (0.98-2.20)	initiate warfarin or antiplatelets	Previous stroke	Hughes et al. (2004) [18]
	
	Asian/Pacific islander	0	Difference = -5.2 (-18.1 to 7.8)	prevalencedifference from non-Hispanic white for receiving warfarin or antiplatelets	Recentischemicstroke	Christian et al. (2003) [17]
		
		0	OR = 0.71 (0.42-1.21)	initiate warfarin or antiplatelets	Previous stroke	Hughes et al. (2004) [18]
		
		-	OR = 0.44 (0.23-0.83)	*discontinue warfarin or antiplatelets*	Previous stroke	Hughes et al. (2004) [18]
	
	Black	-	OR = 0.80 (0.75-0.85)	use of warfarin or antiplatelets	Previous stroke	Quilliam et al. (2001) [12]
	
	Hispanic	0	Difference = - 7.6 (-17.6 to 2.2)	prevalence difference from non-Hispanic white for receiving warfarin or antiplatelets	Recent ischemic stroke	Christian et al. (2003) [17]
		
		0	OR = 0.81 (0.51-1.29)	initiate warfarin or antiplatelets	Previous stroke	Hughes et al. (2004) [18]
		
		0	OR = 1.01 (0.62-1.65)	*discontinue warfarin or antiplatelets*	Previous stroke	Hughes et al. (2004) [18]
	
	Non- Hispanic black	-	OR = 0.62 (0.49-0.78)	initiate warfarin or antiplatelets	Previous stroke	Hughes et al. (2004) [18]
		
		0	OR = 1.03 (0.86-1.24)	*discontinue warfarin or antiplatelets*	Previous stroke	Hughes et al. (2004) [18]
		
		-	Difference = - 7.6 (-11.2 to -3.9)	prevalence difference from non-Hispanic white for receiving warfarin or antiplatelets	Recent ischemic stroke	Christian et al. (2003) [17]
	
	Other	0	OR = 0.95 (0.86-1.04)	use of warfarin or antiplatelets	Previous stroke	Quilliam et al. (2001) [12]
	
	White	0	OR = 0.69 (CI not reported)	use of warfarin or antiplatelets	Previous stroke	Sloane et al. (2004) [13]

Stroke risk	1 risk factor	0	OR = 1.44 (0.52-4.03)	use of warfarin	AF	McCormick et al. (2001) [11]
		
	2 risk factors	0	OR = 2.44 (0.93-6.39)	use of warfarin	AF	McCormick et al. (2001) [11]
		
	3 risk factors	0	OR = 2.37 (0.90-6.20)	use of warfarin	AF	McCormick et al. (2001) [11]
		
	≥ 4 risk factors	0	OR = 2.50 (0.90-6.95)	use of warfarin	AF	McCormick et al. (2001) [11]
		
	High risk	0	OR = 1.49 (0.93-2.36)	use of warfarin	AF	Lau et al. (2004)[10]

AF, atrial fibrillation; CI, confidence interval; OR, odds ratio

#### Previous stroke or transient ischemic attack

Among residents with AF in LTC facilities, both Abdel-Latif et al. [7] (OR = 4.93, 95% confidence interval [CI] = 2.11-11.49 [correction provided by these authors]) and Gurwitz et al. [8] (OR = 1.87, 95% CI = 1.20-2.91), found a positive association between having a history of stroke and receiving warfarin. However, Lau et al. [10] found no significant association between "previous stroke or transient ischemic attack" and warfarin in residents with AF.

#### Atrial fibrillation

Both Quilliam et al. [12] (OR = 2.04, 95% CI = 1.95-2.14) and Hughes et al. [18] (OR = 1.76, 95% CI = 1.50-2.06) found that stroke survivors with AF were twice as likely to receive or be initiated on warfarin or antiplatelet therapy, compared with stroke survivors without AF.

#### Other stroke risk factors

Although both Lau et al. [10] and McCormick et al. [11] evaluated the association between degree of overall stroke risk and use of warfarin in LTC residents with AF, neither study found a significant association. In one study, [12] coronary artery disease was significantly associated (OR = 1.06, 95% CI = 1.02-1.11) with use of warfarin or antiplatelets among stroke survivors, but in another study [10] it was not associated with use of warfarin in residents with AF. While two studies found no significant association between hypertension and use of warfarin in residents with AF [7,10], two other studies found that in stroke survivors hypertension was positively associated with initiation [18] (OR = 1.23, 95% CI = 1.09-1.39) and use [12] (OR = 1.27, 95% CI = 1.22-1.32) of warfarin or antiplatelets.

#### Depression

Both Hughes et al. [18] (OR = 1.22, 95% CI = 1.02-1.46) and Quilliam et al. [12] (OR = 1.11, 95% CI = 1.05-1.18) found that stroke survivors with depression were more likely to initiate or receive warfarin or antiplatelet therapy.

#### Age

Both Gurwitz et al. [8], studying residents with AF (OR = 0.46, 95% CI = 0.22-0.94), and Quilliam et al. [12], studying stroke survivors (OR = 0.86, 95% CI = 0.82-0.91), found that residents ≥ 85 years were less likely to be prescribed either warfarin [8] or warfarin or antiplatelets [12]. However studies of residents with AF [10] and stroke survivors [13,18], respectively, found no significant correlation between age ≥ 85 years and use of these agents. Hughes et al. [18] evaluated stroke survivors in their first year after nursing home admission: residents ≥ 85 years were most likely to discontinue warfarin or antiplatelet therapy (OR = 1.23, 95% CI = 1.05-1.43).

#### Dementia and severe cognitive impairment

Gurwitz et al. [8] reported that AF residents with dementia were less likely than those without it to receive warfarin therapy (OR = 0.59, 95% CI = 0.38-0.90). Quilliam et al. [12] further found that among residents who were stroke survivors, those with Alzheimer's disease (OR = 0.77, 95% CI = 0.70-0.85) or dementia (OR = 0.84, 95% CI = 0.80-0.88) were less likely to receive warfarin or antiplatelets; the likelihood of anticoagulant or antiplatelet therapy also decreased among residents with moderate (OR = 0.93, 95% CI = 0.88-0.97) or severe (OR = 0.63, 95% CI = 0.60-0.67) cognitive impairment [12]. Hughes et al. [18] also found that stroke survivors with severe cognitive impairment were less likely to initiate such therapy (OR = 0.64, 95% CI = 0.52-0.80). In contrast, Sloane et al. [13] found no significant association between severe cognitive impairment and use of warfarin or antiplatelets in stroke survivors.

#### Physical functioning

Both Quilliam et al. [12] (OR = 0.69, 95% CI = 0.64-0.75) and Hughes et al. [18] (OR = 0.73, 95% CI = 0.56-0.96) found that stroke survivors who had dependent physical function were less likely to receive warfarin or antiplatelets than those with independent function. However, Sloane et al. [13] found no significant association between physical dependency and receiving these agents among stroke survivors, and Gurwitz et al. [8] found no significant association between severe physical impairment and the use of warfarin in LTC residents with AF.

#### Bleeding risk factors

Abdel Latif et al. [7] found a negative association (OR = 0.18, 95% CI = 0.03-0.91) between previous gastrointestinal (GI) bleed and warfarin use in residents with AF. Quilliam et al. [12] also found a negative relationship (OR = 0.57, 95% CI = 0.52-0.62) between GI bleeding and warfarin or antiplatelet use in stroke survivors. In that study, Quilliam et al. [12] also found a negative association (OR = 0.64, 95% CI = 0.58-0.71) between peptic ulcer disease and these agents, although Lau et al. [10] found no significant association between peptic ulcer disease and warfarin use in AF. Evaluating bleeding risk factors among AF residents, Lau et al. [10] observed no significant association between warfarin treatment and overall bleeding risk or any single risk factor for bleeding, with the exception of recent surgery. However, McCormick et al. [11] reported that the odds of warfarin treatment were significantly lower (OR = 0.51, 95% CI = 0.29-0.94) for residents with AF and ≥ 2 bleeding risk factors.

#### Race/ethnicity

Among residents with a history of stroke, four studies noted an association between race/ethnicity and being prescribed warfarin or antiplatelet therapy. Christian et al. [17] found that non-Hispanic blacks with a recent hospitalization for ischemic stroke and an indication for warfarin received warfarin less often (7.6% lower rate) than non-Hispanic white residents. In a study by Hughes et al. [18], in the year after nursing home admission, non-Hispanic black stroke survivors were less likely than non-Hispanic whites (OR = 0.62, 95% CI = 0.49-0.78) to be initiated on warfarin or antiplatelet therapy. Quilliam et al. [12] found that black stroke survivors were less likely than whites (OR = 0.80, 95% CI = 0.75-0.85) to receive warfarin or any antiplatelets. Although Abdel-Latif et al. [7] reported that non-Caucasian stroke survivors with AF were less likely than Caucasians to be prescribed warfarin therapy, these findings appear to be bivariate and were not significant in their multivariate model (OR was not reported).

#### LTC facility

For residents within residential care/assisted living facilities, Sloane et al. [13] explored the association between resident and facility characteristics and warfarin or antiplatelet prescribing for stroke survivors. Although bivariate analysis found that several facility characteristics, including larger facilities and those with registered nurses or licensed practical nurses, were associated with non-prescribing, multivariate analysis found no independent association for resident or facility characteristics. After adjusting for facility and resident characteristics, Hughes et al. [18] found that LTC facilities with white-only residents (OR = 0.74, 95% CI = NA) or the presence of full-time physicians (OR = 0.76, 95% CI = 0.63-0.93) were less likely, and that those that were part of a chain (OR = 1.20, 95% CI = 1.01-1.42), were more likely, to initiate warfarin or antiplatelets in stroke survivors. Moreover, facilities with > 10% non-white residents (OR = 1.22, 95% CI = 1.03-1.43), in an urban location (OR = 1.38, 95% CI = 1.16-1.65), having physician extenders on staff (OR = 1.21, 95% CI = 1.0-1.47), or having special care units (OR = 1.33, 95% CI = 1.02-1.73) were more likely to discontinue active warfarin or antiplatelet therapy.

#### Quality assessment

Quality assessment ratings are listed for each of the 14 studies in Table 1. Methodological quality of each study was adequate for all but four areas. One or more limitations or concerns were noted for nine studies [7,9,11,13,17,18,21,24,25]. These included failing to adequately account for potential confounders, failing to consistently describe statistical error for the point estimates reported or to adequately model the relationship between stroke and bleeding risk with warfarin use. In the last case, typical problems noted were either omitting risk factors or else failing to score risks using a validated scoring method or consensus guidelines. Four studies [7,9,17,21] had small sample sizes: subject counts for these studies would not have been able to support a finding of 95% significance for an OR of 1.5. Four studies [17-19,24] had limitations in length of follow-up.

### Prescriber Attitudes and Concerns about Warfarin Use

Two studies (n = 289) [27,29] explored physician attitudes toward warfarin prescribing using case-study questionnaires targeted to physicians (Table 3), while one study (n = 91) [28] (Table 3) explored physician attitudes regarding specialized warfarin services. Dharmarajan et al. [27] evaluated respondent decisions regarding the use of warfarin therapy in the hypothetical case of a white 87-year-old female LTC resident with a history of Alzheimer's disease, surgery for a hip fracture, and AF. The resident, who was wheelchair-bound and dependent for most activities of daily living, had swelling on her forehead from a recent fall, but was negative for fracture on radiological examination. A large majority of responding physicians (85%) believed that long-term warfarin therapy was not indicated for this patient. However, most (88%) said they would prescribe an antiplatelet agent. The reasons most commonly cited for not prescribing warfarin were risk of falls (98%), dementia (40%), and limited life expectancy (32%).

**Table 3:** Prescriber attitudes and concerns with warfarin use

Study	Study objective, (intervention/exposure and outcomes)	Study design, data source	Study population, study setting, time period	Results	Quality assessment, funding source
Dharmarajan et al. (2006) [27]	To evaluate the decision whether or not to anticoagulate among physicians in practice and in various levels of training (residents and fellows) for a specific, yet not unusual, case scenario in the nursing home	*Design*: cross-sectional study *Data source*: survey questionnaire based on an actual case from an LTC facility. The subject of the survey was an 87-year-old female LTC facility resident with dementia, AF, and history of hip fracture who suffered a recent fall without fracture	*Population*: 107 completed surveys were returned from 49 residents, 20 fellows, and 38 attending physicians *Setting*: a university teaching hospital in the Bronx, NY (US) *Time period*: survey dates not specified	The majority of physicians (85%) thought that long-term anticoagulation therapy was not indicated in the case patient. However, most (88%) said they would provide an antiplatelet agent (78% aspirin, 20% clopidogrel). The most cited reasons for not providing anticoagulation were risk of falls (98%), dementia (40%), and short life expectancy (32%). 92% of respondents said the patient was a candidate for short-term anticoagulation therapy. Responses to the questions were similar for all physicians (or faculty) irrespective of level of training or years in practice	*Quality assessment for observational studies:* 1) Unbiased selection of the cohort? Cannot be determined; cohort selection details not provided; non-response rate not disclosed 2) Selection minimizes baseline differences in prognostic factors? Cannot be determined 3) Sample size calculated/5% difference? No 4) Adequate description of the cohort? No; details of cohort other than practice specialty were not provided 5) Validated method for ascertaining exposure? No; reliability concern since limited to 1 case study; content validity of case study not described 6) Validated method for ascertaining clinical outcomes? No; validation assessment of response choices not performed 7) Outcome assessment blind to exposure? NA 8) Adequate follow-up period? Yes; cross-sectional 9) Completeness of follow-up? Yes 10) Analysis controls for confounding? Partial; cross-tabulations performed on responses by specialty 11) Analytic methods appropriate? Yes *Funding*: Geriatric Medicine Fellowship program

Harrold et al. (2002) [28]	To examine physician attitudes regarding the use of specialized anticoagulation services in the LTC setting	*Design*: cross-sectional study *Data source*: survey questionnaire	*Population*: 245 physicians asked to participate in the survey; 114 (47%) responded. 91 reported that they currently cared for residents in LTC facilities and thus completed the questionnaire *Setting*: 21 LTC facilities in Connecticut (US) *Time period*: Nov 1999 - Jan 2000	The majority of respondents agreed or strongly agreed that an anticoagulation service would reduce the workload on physicians (76%), and increase the percent of time that nursing home residents on warfarin are maintained in the target therapeutic range (54%). 53% disagreed or strongly disagreed with statements suggesting that this service would reduce the risk of warfarin-related bleeding. 45% of respondents agreed with a statement that this service would intrude on physician decision-making. 53% of the respondents said they might use an anticoagulation service for managing their LTC patients on warfarin. The most desirable aspects of an anticoagulation service were surveillance for drug interactions (65%), scheduling of laboratory tests (48%), management of warfarin dosing (45%), and risk assessment for bleeding (40%). The most frequently cited challenges to managing warfarin therapy in the nursing home setting were dealing with medications that interact with warfarin (59%), keeping patients within target therapeutic range (53%), and making dosage adjustments (30%)	*Quality assessment for observational studies:* 1) Unbiased selection of the cohort? Yes 2) Selection minimizes baseline differences in prognostic factors? Yes; performed analysis of non-responders 3) Sample size calculated/5% difference? No 4) Adequate description of the cohort? Yes 5) Validated method for ascertaining exposure? No; minimal description of anticoagulation services provided 6) Validated method for ascertaining clinical outcomes? Validation of new questionnaire not reported 7) Outcome assessment blind to exposure? NA 8) Adequate follow-up period? Yes; cross-sectional 9) Completeness of follow-up? Yes 10) Analysis controls for confounding? No; cross tabulation with subject attributes not performed 11) Analytic methods appropriate? No; statistical error (CIs) reported in only some findings *Funding*: Centers for Medicare and Medicaid Services, Department of Health and Human Services; AHRQ

Monette et al. (1997) [29]	To assess the knowledge and attitudes of physicians regarding the use of warfarin for stroke prevention in patients with AF in LTC facilities	*Design*: cross-sectional study *Data source*: survey questionnaire of 2 clinical scenarios with substantial contrasts in patient characteristics: 1) 94-year old male resident with chronic AF, ischemic heart disease, CHF and osteoarthritis, no history of falls, independent in activities of daily living; 2) 80-year old female with recent stroke with resulting hemiplegia and dysarthria, having chronic AF, CHF, CAD, hypertension, diabetes, and chronic renal insufficiency, with cognitive deficits and entirely nonambulatory	*Population*: 269 physicians were asked to participate in the survey; 182 (67.7%) completed the questionnaire *Setting*: 30 LTC facilities located in New England, Quebec, and Ontario (US and Canada) *Time period*: Feb 1995 to Jul 1995	Only 47% of respondents indicated that the benefits of warfarin greatly outweigh the risks in this setting; the remainder of physicians indicated that benefits only slightly outweigh the risks (34%) or that risks outweigh benefits (19%). The most frequently cited contraindications to warfarin use were: excessive risk of falls (71%), history of GI bleeding (71%), history of non-CNS bleeding (36%), and history of cerebrovascular hemorrhage (25%). Among the 164 physicians who reported using the INR to monitor warfarin therapy, 27% indicated a target range with a lower limit < 2.0, 71% indicated a target range between 2.0 and 3.0, and 2% indicated an upper limit > 3.0. Among respondents who answered questions about the clinical scenarios, estimates of the risk of stroke without warfarin therapy and the risk of intracranial hemorrhage with therapy varied widely	*Quality assessment for observational studies:* 1) Unbiased selection of the cohort? Yes 2) Selection minimizes baseline differences in prognostic factors? No; analysis of non-responders was not performed 3) Sample size calculated/5% difference? No 4) Adequate description of the cohort? Yes 5) Validated method for ascertaining exposure? Yes; conducted pre-testing to establish content validity 6) Validated method for ascertaining clinical outcomes? No further assessment validity conducted for new questionnaire 7) Outcome assessment blind to exposure? NA 8) Adequate follow-up period? Yes; cross-sectional 9) Completeness of follow-up? Yes 10) Analysis controls for confounding? Yes 11) Analytic methods appropriate? No; statistical error (CIs) reported in only some findings *Funding*: Dupont Pharma

AF, atrial fibrillation; AHRQ, Agency for Healthcare Research and Quality; CAD, coronary artery disease; CHF, congestive heart failure; CI confidence interval; CNS, central nervous system; GI, gastrointestinal; INR, international normalized ratio; LTC, long-term care; NA not available.

In a similar study, Monette et al. [29] used two case studies - a 94-year old male with chronic AF and co-morbid CHF but having independent physical function and no fall history, and an 80-year old female stroke survivor with AF and CHF who also had hemiplegia, dysarthria, and a recent fall history--to assess physicians' knowledge and attitudes regarding the use of warfarin for stroke prevention in residents with AF. Only 47% of respondents believed that the benefits of warfarin therapy greatly outweighed the risks in this setting; the remainder indicated that benefits outweighed the risks only slightly (34%) or that the risks outweighed the benefits (19%). Contraindications to warfarin use that were cited most frequently included excessive risk of falls (71%), history of GI bleeding (71%), history of other non-central nervous system (CNS) bleeding (36%), and history of cerebrovascular hemorrhage (25%) [29]. Geriatricians were significantly more likely than other physicians to recommend warfarin in the older but more functional case-study resident.

Harrold et al. [28] explored physician attitudes regarding the use of specialized warfarin services in the LTC setting. The majority of physicians agreed that a warfarin service could reduce the workload on physicians and increase the percent of time that residents receiving warfarin are maintained in the target therapeutic range. However, most physicians did not believe that such a service would reduce the risk of warfarin-related bleeding. Only about half of respondents indicated that they might use a warfarin service for managing their residents on warfarin.

#### Quality assessment

Although the three physician surveys described above were innovative, their methodological quality was somewhat low in critical areas. While all three studies introduced new self-administered questionnaires, only one analyzed data regarding non-responders [28], while two [27,28] did not mention steps taken to develop content validity. None of the surveys provided details regarding analysis of item response for the purpose of assessing construct validity. Finally, all three studies had limitations with analysis: failure either to adequately report statistical error or to cross-tabulate responses with subject attributes.

### Warfarin Management and Monitoring

#### International normalized ratio (INR)

The quality of warfarin management and monitoring (Table 4 Part A) was measured in seven studies (n = 10,718) [8,9,11,22,25,30,31], six of which (n = 10,681) [8,9,11,22,25,30] evaluated the percentage of time or person-days spent by residents in therapeutic range, based on INR results. In these six studies, INR levels were in the target range (2.0-3.0) for a mean 48% of the time (range = 37-55%). Four of these studies [8,11,22,25] also reported percentage of time or person-days in subtherapeutic or supratherapeutic ranges. INR levels were in the subtherapeutic range (< 2.0) for a mean of 38% of the time (range = 35-45%) and in the supratherapeutic range (> 3.0) for a mean of 14% of the time (range = 11-16%). One study, Karki et al. [31], reported only mean INRs for residents and only for those whose INR values indicated either adequate or poor INR control.

**Table 4:** Warfarin management and monitoring

Study	Study objective, (intervention/exposure and outcomes)	Study design, data source	Study population, study setting, time period	Results	Quality assessment, funding Source
**Part A: Quality of oral anticoagulant prescribing and monitoring**					

Aspinall et al. (2010) [15,30]	To describe the quality of warfarin prescribing and monitoring in VA nursing homes and to assess factors associated with maintaining a therapeutic INR	*Design:* *retrospective cohort study* *Data source: *medical record review	*Population: *all veterans (160) who received warfarin *Setting: *5 VA nursing homes (US) *Time period:* Jan 1, 2008 - June 30, 2008	INRs were in therapeutic range for 55% of the 10,380 total person-days of warfarin. In a 4-week period, patients had an average of 5.2 (SD = 2.7) INRs obtained. 99% of the INR tests were repeated within 4 weeks of the previous result. 49% of patients had INRs in the target range for ≥ 50% of their person-days. Achieving this outcome was more likely in patients with prevalent warfarin use than with new use (adjusted OR = 2.86; 95% CI = 1.06-7.72). Patients with a history of a stroke (adjusted OR = 50.38; 95% CI = 50.18-0.80) were less likely to have therapeutic INRs for > 50% of their days. Approximately 89% of the patients at baseline were receiving ≥ 1 medication that potentially interacts with warfarin. The most frequently prescribed interacting drugs at baseline were omeprazole (51% of patients), simvastatin (45%), aspirin (34%), citalopram (18%), and levothyroxine (13%). During the study period, 46% of patients were prescribed a medication with the potential to interact with warfarin	*Quality assessment* *for observational studies:* 1) Unbiased selection of the cohort? Yes 2) Selection minimizes baseline differences in prognostic factors? Yes 3) Sample size calculated/5% difference? No 4) Adequate description of the cohort? Yes 5) Validated method for ascertaining exposure? Yes 6) Validated method for ascertaining clinical outcomes? Yes 7) Outcome assessment blind to exposure? Yes 8) Adequate follow-up period? Partial; length of follow-up not clearly stated in Methodology 9) Completeness of follow-up? Yes 10) Analysis controls for confounding? Yes 11) Analytic methods appropriate? Yes *Funding: *various VA centers and US government agencies

Gurwitz et al. (2007) [25] (repeated from table 1)				The percentages of time in the < 2, 2-3, and > 3 INR ranges were 36.5%, 49.6%, and 13.9%, respectively	

Gurwitz et al. (1997) [8] (repeated from table 1)				Of 122 warfarin users with adequate INR data, warfarin therapy was monitored at least every 2 weeks in 52% of the subjects, every 2-4 weeks in 32% of the subjects, and less frequently than every 4 weeks in only 16% of the subjects. On average, 117 NVAF residents with available INR data were maintained in the therapeutic range 39.6% of the time, in the subtherapeutic range 44.8% of the time, and in the supratherapeutic range 15.6% of the time; < 23 subjects (20%) were in the therapeutic range ≥ 60% of the time	

Karki et al. (2003) [31]	To evaluate the warfarin management patterns in an academic nursing home and evaluate what pre-determined factors are associated with variability in the INR	*Design*: case control study *Data source*: medical chart review	*Population*: 37 residents receiving warfarin therapy for > 3 consecutive months in a calendar year *Setting*: 566-bed academic medical center nursing home (US) *Time period*: 12-month period; dates not specified	For patients who had INR values exceeding the therapeutic range there was no significant difference between "easy" management (INR fluctuations of 0.5-0.99 and outside therapeutic range ≤ 10% of time, n = 18) and "difficult" management (with INR fluctuations > 0.99 and outside therapeutic range > 10% of time, n = 19) in all factors examined. The "difficult management" group received more medications known to interact with warfarin than the "easy" management. These medications may have caused the INR to increase above the normal range (*P*=0.003), as well as produced large (*P*=0.001) or small fluctuations (*P*=0.0007) in the INR. 54% of residents on warfarin therapy initiated a potential warfarin-interacting drug. Of all interacting medications, 55% were antibiotics and 28% were analgesics	*Quality assessment* *for observational studies:* 1) Unbiased selection of the cohort? Partial; limited to residents of a single nursing home 2) Selection minimizes baseline differences in prognostic factors? Yes 3) Sample size calculated/5% difference? No; very low power 4) Adequate description of the cohort? No; only limited description of nursing home and resident characteristics reported 5) Validated method for ascertaining exposure? No; assignment of subjects to "easy management" and "difficult management" cohorts not validated 6) Validated method for ascertaining clinical outcomes? Yes 7) Outcome assessment blind to exposure? Yes 8) Adequate follow-up period? Yes 9) Completeness of follow-up? Yes 10) Analysis controls for confounding? Partial; evaluated association of age, gender, and number of illnesses with cohort 11) Analytic methods appropriate? Partial; only limited univariate analyses conducted *Funding*: not stated

Lackner et al. (1995)[9] (repeated from table 1)				The INR was within the recommended range for NVAF over a 6-month period 37% of the time and recommended PT, 52% of the time. An equal percentage of warfarin dose changes occurred in response to a PT ratio outside the recommended range as occurred with an INR outside the recommended range	

McCormick et al. (2001) [11] (repeated from table 1)				In the 42% of AF patients who were receiving warfarin therapy, the therapeutic range of INR values was maintained only 51% of the time, was below the therapeutic range 36% of the time, and was above the therapeutic range 13% of the time	

Verhovsek et al. (2008) [22]	To determine how effectively warfarin was administered to a cohort of residents in LTC facilities by measuring TTR, to identify the proportion of residents prescribed warfarin-interacting drugs and to ascertain factors associated with poor INR control	*Design*: Retrospective cohort study *Data source*: medical chart review	*Population*: 105 LTC residents receiving warfarin therapy *Setting*: 5 LTC facilities in Hamilton, Ontario (Canada) *Time period*: 12 months of data for each resident between October 2004 and April 2005	3065 INR values were available. Residents were within, below, and above the therapeutic range 54%, 35% and 11% of the time, respectively. 79% of residents were prescribed ≥ 1 warfarin-interacting medication during the period in review. The 5 most common drugs were acetaminophen (40% of residents), citalopram (25%), acetylsalicylic acid (16%), diltiazem (11%), and simvastatin (10%). Residents receiving interacting medications spent less TTR (53.0% vs 58.2%, OR = 0.93; 95% CI = 0.88-0.97, *P*=0.002). Adequacy of anticoagulation varied significantly between physicians (TTR range 45.9-63.9%)	*Quality assessment* *for observational studies:* 1) Unbiased selection of the cohort? Yes 2) Selection minimizes baseline differences in prognostic factors? Yes 3) Sample size calculated/5% difference? No; low power for both residents and physicians studied 4) Adequate description of the cohort? Yes 5) Validated method for ascertaining exposure? Yes 6) Validated method for ascertaining clinical outcomes? Yes 7) Outcome assessment blind to exposure? Yes 8) Adequate follow-up period? Partial; some residents may have had < 6 month follow-up of INR 9) Completeness of follow-up? Yes 10) Analysis controls for confounding? No; association of INR outcomes with subject attributes not analyzed 11) Analytic methods appropriate? No; analysis of residents limited to simple one-way tabulations *Funding*: CIHR and the Regional Medical Associates

**Part B: Medication management interventions**					

Allen et al. (2000) [23]	To evaluate the effectiveness of nurse practitioner management of anticoagulation using a protocol. Outcomes were frequency of blood draws as well as frequency and percentage of INRs that were out of range	*Design*: retrospective cohort study *Data source*: nurse practitioner maintained records	*Population*: 47 patients on long-term anticoagulation therapy *Setting*: 9 area nursing homes (US) *Time period*: 6 months beginning June 1997	Average number of venipuncture ranged from 0.7 -2.7 per month. Reasons for out-of-range INRs were identified 35% of the time. Percentage out of range was 15%	*Quality assessment* *for observational studies:* 1) Unbiased selection of the cohort? Cannot be determined; convenience sample of 47 residents 2) Selection minimizes baseline differences in prognostic factors? Yes 3) Sample size calculated/5% difference? No 4) Adequate description of the cohort? No; resident characteristics not described 5) Validated method for ascertaining exposure? Yes 6) Validated method for ascertaining clinical outcomes? Yes 7) Outcome assessment blind to exposure? No 8) Adequate follow-up period? Cannot be determined; post-intervention follow-up period not described 9) Completeness of follow-up? Cannot be determined 10) Analysis controls for confounding? No; no evaluation of association of resident attributes with INR outcomes 11) Analytic methods appropriate? No; Statistical error not reported *Funding*: not specified

Papaioannou et al. (2010) [24] (repeated from Table 1)				Overall, TTR increased during the MEDeINR phase (65-69%), but was significantly increased for only 1 facility (62-71%, *P*< 0.05). The percentage of time in supratherapeutic range decreased from 14% to 11%, *P*=0.08); there was little change for the subtherapeutic range (21% to 20%, *P*=0.66). Overall, the average number of INR tests per 30 days decreased from 4.2 to 3.1 (*P*< 0.0001) per resident post-implementation. Feedback from LTC clinicians and staff indicated that the program decreased workload, improved confidence in management and decisions, and was generally easy to use	

CI, confidence interval; CIHR, Canadian Institute of Health Research; INR, international normalized ratio; LTC, long-term care; NVAF, nonvalvular atrial fibrillation; OR, odds ratio; PT, prothrombin time; SD, standard deviation; TTR, time in therapeutic range; VA, Veteran's Administration

Two studies that evaluated the effectiveness of warfarin medication management systems [23,24] (n = 175, Table 4 Part B) found post-interventional time in therapeutic range considerably higher than reported in the six studies above. Papaioannou et al. [24] studied the effects of an electronic decision-support system for warfarin dosing and found that time in therapeutic range was 65% (pre-intervention) to 69% (post-intervention; the post-interventional change was non-significant). In a study of warfarin management by nurse practitioners, Allen et al. [23] measured 85% of INR draws within therapeutic range. These measurements were post-intervention only; no pre-intervention or control results were reported.

#### Factors associated with INR range

Three studies [22,30,31] (Table 4 Part A) evaluated the association of resident characteristics with INR (n = 302). Aspinall et al. [30] noted that residents who began warfarin therapy before the study period and those who did not have a history of stroke were more likely to maintain INR values within the therapeutic range. Karki et al. [31] observed that a group of LTC residents with INR fluctuations > 0.99 between blood samplings received more medications known to interact with warfarin vs a group with INR fluctuations ranging from 0.5-0.99. Verhovsek et al. [22] found that residents receiving interacting medications spent less time in therapeutic range. Time in therapeutic range varied significantly among prescribing physicians [22].

#### Potential warfarin interactions

The three studies cited above [22,30,31] (Table 4 Part A) also evaluated the use of potentially interacting medications among residents receiving warfarin for any condition (n = 302). Aspinall et al. [30] reported that 89% of residents were receiving a medication that potentially interacts with warfarin. The most common agents were omeprazole (51% of residents), simvastatin (45%), aspirin (34%), citalopram (18%), and levothyroxine (13%). Karki et al. [31] reported that 46% of LTC residents were prescribed a potentially interacting agent during the study period. Verhovsek et al. [22] found that 79% of residents were prescribed more than one warfarin-interacting medication during a 12-month period; most common were acetaminophen (40% of residents), citalopram (25%), aspirin (16%), diltiazem (11%), and simvastatin (10%).

#### Quality assessment

Overall, in the nine warfarin management and monitoring studies described above (Table 4 Parts A and B), methodological quality was low to moderate. Low power was common: five studies [9,22,23,30,31] had an insufficient number of subjects to find 95% significance for an odds ratio of 1.5. Four studies [22-24,30] had either inadequate descriptions of resident follow-up or insufficient follow-up periods to achieve the stated study purpose. Seven studies [9,11,22-25,31] had deficiencies either in adequately analyzing or reporting study findings or in accounting for potential confounders.

### Warfarin-related Adverse Events

Two studies [25,26] (Table 5) sought to evaluate adverse warfarin-related events in the LTC population (n = 17,429). In a study of 25 nursing homes, Gurwitz et al. [25] determined the combined overall rate of adverse warfarin-related events (18.8 per 100) and potential adverse warfarin-related events (6.6 per 100) to be 25.5 per 100 resident-months on warfarin therapy. Of the adverse warfarin-related events, 11% were deemed serious and 2% were life-threatening or fatal. Overall, 29% of the adverse warfarin-related events were considered preventable; of the serious, life-threatening, and fatal events, over half (57%) were considered preventable. Quilliam et al. [26] explored an association between warfarin and the antiplatelet agents (excluding clopidogrel, which was not yet available) and risk of hospitalization for bleeding among LTC residents. After adjustment for demographic characteristics and medications, use of warfarin, compared to no warfarin or antiplatelet medication, was associated with a significantly increased risk of hospitalization for bleeding. Recipients of warfarin plus aspirin were more likely to be hospitalized for a CNS bleeding event; recipients of warfarin alone were more likely to be hospitalized for a GI bleeding event.

**Table 5:** Warfarin-related adverse events

Study	Study objective, (intervention/exposure and outcomes)	Study design, data source	Study population, study setting, time period	Results	Quality assessment, funding source
Gurwitz et al. (2007) [25] (repeated from Table 1)				720 warfarin-related AEs and 253 potential warfarin-related AEs were identified. Of the warfarin-related AEs, 87% were characterized as minor, 11% were deemed serious, and 2% were life-threatening or fatal. Overall, 29% of warfarin-related AEs were judged to be preventable. The rate of warfarin-related AEs was 18.8 per 100 resident-months on warfarin therapy (95% CI, 17.5-20.3 per 100 resident-months), with a rate of 5.4 preventable warfarin-related AEs per 100 resident-months (95% CI, 4.7-6.2 per 100 resident-months). Potential warfarin-related AEs occurred at a rate of 6.6 per 100 resident-months on warfarin (95% CI, 5.8-7.5 per 100 resident-months). Serious, life-threatening, or fatal events occurred at a rate of 2.5 per 100 resident-months (95% CI, 2.0-3.0 per 100 resident-months); 57% of these more severe AEs were considered preventable. Errors resulting in preventable AEs occurred most often at the prescribing and monitoring stages of warfarin management	

Quilliam et al. (2001) [26]	To quantify the effect of antiplatelet and anticoagulant agents on risk of hospitalization for bleeding among an elderly nursing home stroke survivors	*Design: *case- control study *Data source:* Medicare claims and MDS assessments in the SAGE database	*Population: *nursing home residents, 3433 cases (residents with first hospitalization for bleeds) and 13,506 controls (residents having no such hospitalizations) *Setting: *nursing homes in5 states (US) *Time period: *1992-1997	After adjustment, use of warfarin (OR = 1.26; 95% CI = 1.11-1.43) and combination (antiplatelet and warfarin) therapy (OR = 1.34; 95% CI = 0.99-1.82) were associated with an increased risk of hospitalization for a bleed compared with nonusers. The odds of aspirin use was greater among cases than controls (OR, 1.07; 95% CI = 0.96-1.18) after adjustment. The numbers needed to treat to seriously harm (e.g. hospitalization for a bleed) 1 resident with aspirin and warfarin were 467 and 126, respectively. The odds of a CNS bleed with aspirin use was 1.36 (95% CI = 1.05-1.78) and 1.64 (95% CI = 1.19- 2.26) for warfarin use. The number needed to treat for harm values for CNS; bleeds were 534 (95% CI = 214- 3846) for aspirin and 301 (95% CI = 153-1012) for warfarin. Patients with GI bleeding were more likely to have taken warfarin (OR = 1.18; 95% CI = 1.03-1.36); number needed to treat for harm, 228 (95% CI = 114- 1366). Aspirin users were not more likely to be hospitalized for GI bleeds (OR = 1.01; 95% CI = 0.91- 1.14)	*Quality assessment for observational studies:* 1) Unbiased selection of the cohort? Yes 2) Selection minimizes baseline differences in prognostic factors? Yes 3) Sample size calculated/5% difference? Yes 4) Adequate description of the cohort? Yes 5) Validated method for ascertaining exposure? Yes 6) Validated method for ascertaining clinical outcomes? Yes 7) Outcome assessment blind to exposure? Yes 8) Adequate follow-up period? Yes; cross-sectional 9) Completeness of follow-up? Yes 10) Analysis controls for confounding? Yes 11) Analytic methods appropriate? Yes *Funding: *AHRQ

AEs, adverse events; AHRQ, Agency for Healthcare Research and Quality; CI, confidence interval; OR, odds ratio; MDS, Minimum Data Set; SAGE, Systematic Assessment of Geriatric drug use via Epidemiology

#### Quality assessment

Except for limitations in one study, which neither adequately controlled for confounding nor explored the association of poor INR control with warfarin-related adverse events or resident characteristics [25], methodological quality for these two adverse event studies was acceptable.

## Discussion

Results from this review show that AF, DVT, PE, and stroke prevention in residents without AF, account for nearly all warfarin use in the LTC setting [24,25]. Of these four, AF is the primary indication for warfarin use, accounting for the majority, or as many as three of every four residents receiving this agent in LTC [24,25].

### Suboptimal anticoagulation among patients with AF

Current consensus guidelines, including those of the American College of Chest Physicians (ACCP) [32-34], American College of Cardiology/American Heart Association/European Society of Cardiology (ACC/AHA/ESC) [35], and the ESC alone [36], strongly support the use of warfarin in patients with AF who have one or more high risk factors or two or more moderate risk factors for stroke, especially among those not at high risk for bleeding. The American Medical Directors Association (AMDA) guidelines for stroke management in LTC, updated in 2011, also recommend warfarin as primary therapy for stroke prophylaxis in AF, but suggest that a systematic assessment of stroke and bleeding risk be performed prior to initiation of therapy [2]. Lau et al. in this review found that nearly all LTC residents with AF were at high risk for stroke simply because 88% of residents were ≥ 75 years of age[10].

Current AMDA guidelines cite a general low use of warfarin in residents with AF who have no contraindications for its use [2]. Evidence from five LTC studies in our review suggests a pattern of improving but still low rates of warfarin use in AF: among LTC residents with AF studied since 1997, between 46% and 60% were prescribed warfarin [7,10,11]; in earlier studies dating to the mid-1990s, only 17% to 32% received warfarin [8,9]. Our findings suggest that bleeding risk alone may not explain the low rates of warfarin use for AF in LTC. Even among candidates described as having high stroke risk and low bleeding risk, rates of warfarin use among LTC residents with AF in three studies were 20% [10], 53% [11], and 60% [10].

### Physician concerns illustrate resistance to using warfarin

Physician concerns may, in part, explain low observed warfarin use in the LTC setting. Surveys of physicians using case-study examples showed that respondents strongly supported use of antiplatelet agents [27]. However, these same respondents appeared reluctant to prescribe warfarin for the case-study residents, each of whom had AF plus one or more major risk factors for stroke. Only half of surveyed physicians agreed that benefits of warfarin therapy greatly outweigh the risks [29]. Specific concerns motivating physicians to avoid using warfarin seem clear, starting with risk of falls in two studies (71% and 98%) [26,28], and including dementia [27], short life expectancy [27], history of GI bleeding [29], history of other non-CNS bleeding [29], and history of cerebrovascular hemorrhage [29].

### Consensus noted among some factors associated with use of warfarin

In this review, several factors were associated with greater use of stroke prevention therapy--AF in stroke survivors [12,18], history of stroke [7,8] in residents with AF, hypertension [12,18] in stroke survivors, and depression [12,18] in stroke survivors. Dementia and severe cognitive impairment were generally associated with lower use or initiation of stroke prevention therapy in LTC [8,12,18]. Previous GI bleeding was also strongly and negatively linked to use of warfarin or antiplatelets in patients with AF and in stroke survivors [7,12].

This review also found that black race was generally associated with lower, adjusted use of therapy. Although one study [7] did not find a significant multivariate association between African-American race and use of warfarin or antiplatelets in stroke survivors, the number of observed subjects (n = 117) may have been too small to adequately test this factor. Three other studies [12,17,18] that examined the same therapies in strongly powered multivariate models found evidence of a significantly lower use of these agents among black compared with white stroke survivors.

Conflicting findings or no evidence of association were found across studies for other resident characteristics and use of stroke prevention therapy: age ≥ 85 years (reduction or no association), coronary artery disease (increase or no association), dependent physical functioning (reduction or no association), peptic ulcer disease (reduction or no association), high overall bleeding risk (reduction or no association), and overall stroke risk (no association).

### Warfarin management and monitoring signal that INR levels remain difficult to maintain

Studies in this review consistently showed that in LTC residents receiving warfarin, INRs are in the target range approximately half of the time [8,9,11,22,25,30]. With further evidence that only one in five residents exceed 60% of monitored time in therapeutic range [8], achieving INR targets is a potentially serious problem in the LTC setting. Comparing mean values reported in this review, residents who fall out of target range are 2.5-3 times more likely to have INR levels that are subtherapeutic, which exposes them to a potentially greater risk of stroke, rather than supratherapeutic, which would increase their bleeding risk. Studies in this review suggest that blood draws in LTC occur frequently [8,30], at least once per month for 84% or more of residents [8,30], and more often for many residents [23,24]. McCormick et al. [11] discuss the seeming paradox in the controlled-care environment of LTC where such INR control problems often occur. Although non-adherence to medication or INR monitoring poses fewer problems in the LTC setting, and despite potentially better control of interacting medications and diet, warfarin prescribing and INR monitoring appear less than optimal [11].

Several factors may be associated with reduced time in INR therapeutic range. These include history of stroke [30] and medication interactions [22,31]. Regarding the latter, evidence from this review suggests that the opportunity for warfarin interactions in the LTC facility is great, with 46-89% [22,30,31] of residents taking a potentially interacting medication.

The two studies that evaluated medication management systems [23,24] reported substantially higher post-intervention times in target range than those discussed above. However, in one of these studies [24], the use of an electronic decision support system for warfarin dosing produced no significant gain across facilities in time-in-range when compared to the pre-intervention period. Findings from the second study [23] showed that 85% of post-intervention INR draws were within range; however, these results are largely uninterpretable, since the study lacked pre-intervention data or a separate control group.

Although no study to date has established a link between poor INR control and health outcomes in LTC, warfarin-related adverse events are common in the LTC facility: the combined overall rate of adverse warfarin-related events and potential adverse warfarin-related events was high, equivalent to one adverse effect for every 4 months on therapy [25]. The use of warfarin was also associated with a significantly increased risk of hospitalization for a bleeding event [26].

## Conclusions

Consensus guidelines regarding the use of warfarin for primary or secondary stroke prevention in AF may not be sufficiently followed in the LTC setting, where warfarin use appears suboptimal. The challenges of warfarin use in the LTC setting are apparent to physicians and, in their view, may outweigh the benefits of stroke risk reduction. For LTC residents who are receiving warfarin, maintaining therapeutic INR remains problematic, even in the highly controlled LTC environment. Further research is needed to evaluate appropriate use of anticoagulants in this setting, including a more explicated consideration of the appropriate balancing of risks and benefits in an important but problematic issue for the geriatric clinician.

## Competing interests

AAP and WWN are employees of Janssen Scientific Affairs, LLC (a Johnson and Johnson company) and shareholders of Johnson and Johnson. At the time this research was conducted, MN and GR were consultants to Janssen Scientific Affairs, LLC and received funding for this research and writing of the review manuscript. Janssen Scientific Affairs, LLC separately provided financial support for editing services and for the article processing charge associated with this author-prepared manuscript. Over the past five years GR also received funding from Pfizer Inc. for contracted research and services and development of other manuscripts. The authors declare no other financial or non-financial competing interests.

## Authors' contributions

Coauthors' contributions to this manuscript include the following: MN (study design, article search and summarization, table production, analysis, manuscript production), AAP (initial study concept, study design, manuscript rewrite), WWN (study design, critical review, manuscript rewrite), GR (study design, article search and summarization, table production, analysis, manuscript production). All authors read and approved the final manuscript.

## Pre-publication history

The pre-publication history for this paper can be accessed here:

http://www.biomedcentral.com/1471-2318/12/14/prepub
